# Mesenchymal stem cells-derived extracellular vesicles ameliorate Alzheimer’s disease in rat models via the microRNA-29c-3p/BACE1 axis and the Wnt/β-catenin pathway

**DOI:** 10.18632/aging.203088

**Published:** 2021-06-04

**Authors:** Sha Sha, Xueli Shen, Yunpeng Cao, Le Qu

**Affiliations:** 1 Department of Geriatrics, The First Affiliated Hospital of China Medical University, Shenyang 110001, Liaoning Province, China; 2 Department of Neurology, The First Affiliated Hospital of China Medical University, Shenyang 110001, Liaoning Province, China; 3 Department of Dermatology, The First Affiliated Hospital of China Medical University, Shenyang 110001, Liaoning Province, China

**Keywords:** Alzheimer's disease, bone marrow mesenchymal stem cells, extracellular vesicles, microRNA-29c-3p, BACE1

## Abstract

Currently, Alzheimer’s disease (AD) cannot be treated effectively. Mesenchymal stem cells (MSCs)-derived extracellular vesicles (EVs) (MSC-EVs) exhibit therapeutic effects on many diseases. This study investigated the mechanism of bone marrow MSC-EVs (BM-MSC-EVs) in a rat model of AD. The cognitive function, amyloid-β (Aβ) plaques, Aβ deposition areas and levels of Aβ1-42, Aβ decomposition-related factors (NEP and IDE), and inflammatory cytokines in BM-MSC-EVs-treated AD rats were measured. The effect of BM-MSC-EVs was studied in AD neuron model. microRNA (miR)-29c-3p and BACE1 expression, as well as levels of Wnt/β-catenin pathway-related factors in AD and EVs-treated AD models were detected. miR-29c-3p relationship with BACE1 was predicted and confirmed. miR-29c-3p and BACE1 were interfered to verify the mechanism of EVs in AD. The Wnt/β-catenin pathway inhibitor DKK1 was further added to EVs-treated AD neurons. BM-MSC-EVs showed therapeutic effects on AD rats and neurons. BM-MSC-EVs carried miR-29c-3p into AD neurons. miR-29c-3p targeted BACE1. Silencing miR-29c-3p in BM-MSCs reduced BM-MSC-EV therapeutic effect on AD, which was reversed after BACE1 knockdown. miR-29c-3p targeted BACE1 and activated the Wnt/β-catenin pathway, and the Wnt/β-catenin pathway inhibition impaired EV therapeutic effects on AD. We highlighted that BM-MSC-EVs delivered miR-29c-3p to neurons to inhibit BACE1 expression and activate the Wnt/β-catenin pathway, thereby playing a therapeutic role in AD. This study may provide a novel perspective for elucidating the mechanism of MSCs in the treatment of AD.

## INTRODUCTION

Alzheimer’s disease (AD), a complex progressive neurodegenerative disorder, is the most common type of dementia worldwide and frequently accompanied by memory impairment, cognitive decline, disorientation, and even death [1, 2]. Pathologically, AD is predominately characterized by the formation of amyloid-beta (Aβ) plaques and neurofibrillary tangles, together with the subsequent loss of neurons [3]. Inflammation has been shown to be involved in the early stages of AD pathogenesis [4]. It remains challenging to characterize AD, and a vast majority of patients are not diagnosed until the middle or advanced stages when the brain has shown irreversible damage [5]. Currently, despite the certain efficacy of six approved drugs for AD treatment, they only relieve symptoms and fail to intervene AD pathologically [6]. Therefore, developing more effective approaches for AD therapy is of great urgency.

Recently, extracellular vesicles (EVs), nanometer-sized cell-secreted membrane vesicles, have been proven to play a critical role in neurodegenerative diseases, and EVs-based therapy may present with tremendous clinical benefits [7, 8]. Almost all cell types can secrete EVs, and among them, mesenchymal stem cells (MSCs)-derived EVs (MSC-EVs) are the most promising due to their easy accessibility and maintenance, which have been found to play an amazing therapeutic role in neurodegeneration [9, 10]. MSC-EVs have been validated as effective therapeutic delivery tools for AD [11]. MSC-EVs suppress iNOS expression and alleviate neural impairment in AD mice [12]. MSC-EVs counteract neuroinflammation and enhance antioxidant capacity, thus serving as a potential therapy to retard Parkinson's disease progression and ameliorate symptoms [13]. It is well established that EVs shuttle their functional cargoes such as proteins, microRNAs (miRs), and phospholipids for intercellular communication [14]. It has shown that MSC-EVs exert effects on brain diseases via the transfer of their carried miRs [15]. miRs, a class of short noncoding RNAs, are tightly involved in AD pathogenesis, showing the strong potential of being therapeutic biomarkers for AD [16]. miR-29c-3p has been proposed to be a helpful biomarker for AD with its downregulated expression [17]. Also, previous literature has demonstrated that miR-29c-3p expression can be detected in MSC-derived EVs and exert cardioprotective effects [18]. However, the underlying interplay between MSC-EVs and miR-29c-3p in AD has not been fully elaborated.

Therefore, we took the initiative to speculate that bone marrow MSC-EVs (BM-MSC-EVs) may play a role in AD with the involvement of miR-29c-3p. Consequently, we performed a series of histological and molecular experiments to identify the effects of BM-MSC-EVs on AD, and to explore the related regulatory mechanism, in order to provide some novel therapies against AD.

## RESULTS

### BM-MSC-EVs were successfully isolated

MSCs have been used to treat the animal model of AD and other neurodegenerative diseases [19–22]. Recent studies have identified EVs as important mediators of cell signal communication [23, 24]. BM-MSC-EVs have been shown to play a regulatory role in a variety of diseases [25, 26]. BM-MSCs were firstly extracted from SD rats and observed 24 h later under an inverted microscope. Some cells adhered to the wall with round shape and varied size. After 4-5 days of culture, the number of adherent cells was gradually increased, and polygonal or spindle-shaped cell colonies were formed. On the 10^th^ day, the cells were passaged when growing to 90% confluence. BM-MSCs of the 2^nd^ generation were mainly spindle-shaped with vortex distribution, showing typical morphologies of BM-MSCs (Supplementary Figure 1A). Flow cytometry showed that the surface markers CD29 (97.78%) and CD90 (78.21%) were highly expressed, while the hematopoietic stem cell markers CD34 (0.078%) and CD45 (0.79%) were poorly expressed (Supplementary Figure 1B) on BM-MSCs of the 3^rd^ generation. In addition, BM-MSCs were induced for osteogenic and adipogenic differentiation, and their osteogenic/adipogenic differentiation potentials were verified (Supplementary Figure 1C, 1D). The above results indicated that BM-MSCs were successfully isolated.

Additionally, BM-MSC-EVs were identified as disc-shaped bilayer membrane structures with round and oval shape and a diameter of 50-100 nm under a TEM (Supplementary Figure 1E). According to WB results, EVs surface markers CD9 and CD63 were expressed in BM-MSC-EVs, while endoplasmic reticulum marker calnexin showed no expression in BM-MSC-EVs (Supplementary Figure 1F). NTA revealed that the main particle size of BM-MSC-EVs ranged from 30-100 nm, with a median of 90 nm (Supplementary Figure 1G). From all above, we confirmed that BM-MSC-EVs were successfully extracted.

### BM-MSC-EVs had therapeutic effects on AD rats

**Figure 1:**
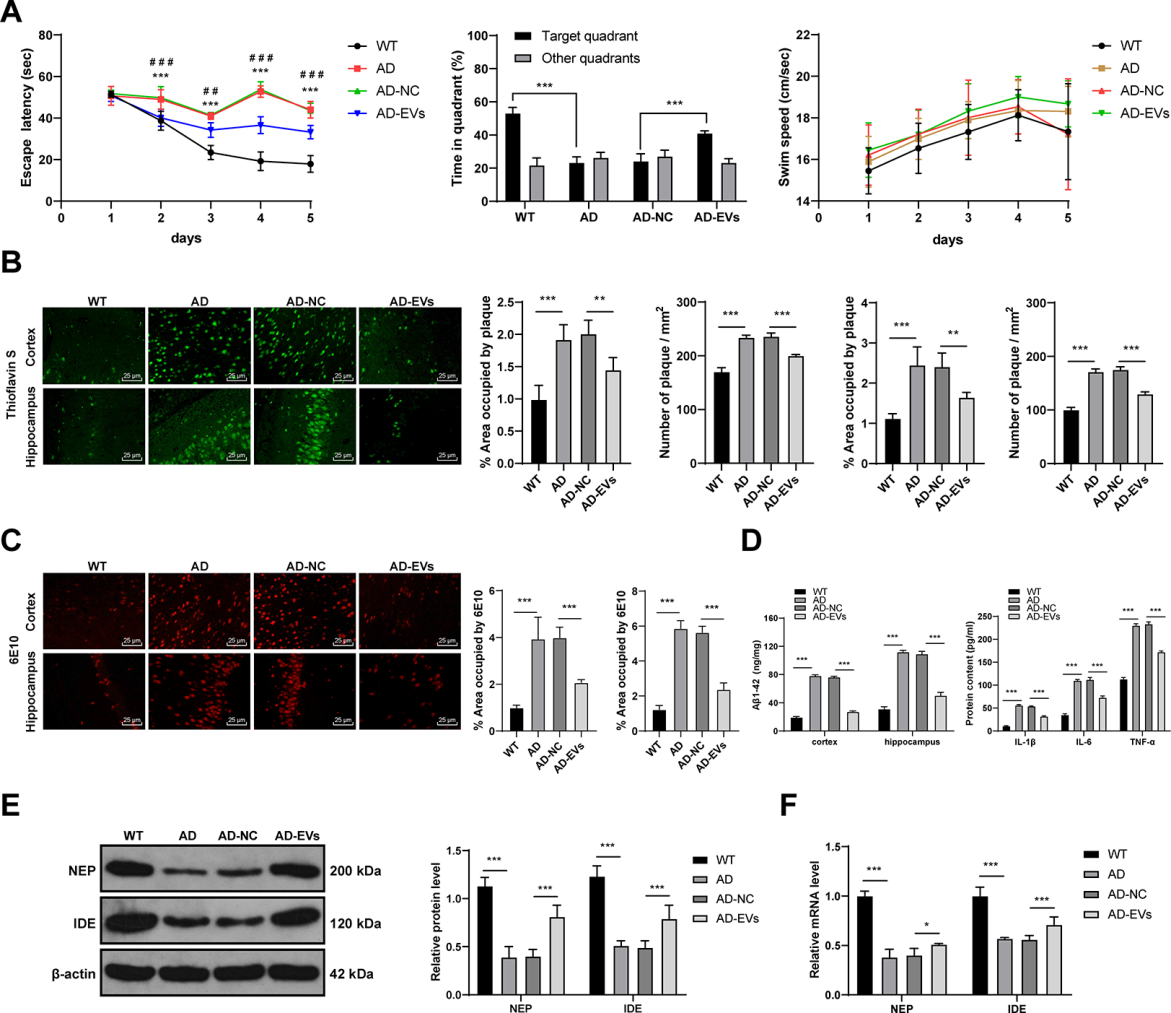
**BM-MSC-EVs have therapeutic effects on AD rats.** The rat model of AD was established by injection of Aβ1-42, and then rats were treated with BM-MSC-EVs, with injection of equal volume of BM-MSC conditioned medium after GW4869 treatment as the control. (**A**) Rat behaviors and memory abilities were measured using Morris Water maze test; (**B**) Thioflavin S staining for Aβ deposition in cerebral cortex and hippocampus of rats in each group; (**C**) Immunofluorescence assay was used to detect Aβ content in cerebral cortex and hippocampus of rats in each group; (**D**) ELISA was used to detect Aβ1-42 level in cerebral cortex and hippocampus and levels of inflammatory cytokines (IL-1β, IL-6 and TNF-α) in cerebral tissues of rats in each group; (**E**) WB was used to detect the protein levels of NEP and IDE; (**F**) RT-qPCR was used to detect the mRNA expression of NEP and IDE. n = 6. Data were expressed as mean ± standard deviation. Data were analyzed using one-way ANOVA followed by Tukey’s multiple comparisons test. In panel (**A**) ***p < 0.001 (WT vs AD), ### *p* < 0.001 (AD-NC vs AD-EVs). In other panels, *p < 0.05, **p < 0.01, ***p < 0.001.

The rat model of AD was established by intracerebroventricular injection of Aβ1-42 to study the therapeutic effects of BM-MSC-EVs on AD rats. Firstly, the Morris water maze test was used to test rat motor ability. We found that AD rats showed remarkably reduced motor ability (all *p* < 0.001) with no difference in swimming speed (Figure 1A). Given that Aβ deposition is a typical AD pathology, we stained the rat cerebral tissues with Thioflavin S. AD rats exhibited obviously more Aβ deposition area and Aβ plaques than WT rats (all *p* < 0.001) (Figure 1B). In addition, 6E10 antibody was further used for Aβ deposition staining [27], and similar results to those in Thioflavin S staining were observed (all *p* < 0.001) (Figure 1C). According to ELISA results, levels of Aβ1-42 and inflammatory cytokines (IL-1β, IL-6, and TNF-α) in AD rat cerebral tissues were notably increased (all *p* < 0.001) (Figure 1D). Furthermore, as shown by RT-qPCR and WB results, the expressions of NEP and IDE (two major Aβ-degrading enzymes) were obviously decreased (Figure 1E, 1F). These results indicated that the AD rat model was successfully induced.

Subsequently, the therapeutic effects of BM-MSC-EVs on AD were detected. It was observed that EV treatment remarkably reduced escape latency of AD rats and increased the percentage of time in the target quadrant (all *p* < 0.001), with no change in the swimming speed (Figure 1A). Additionally, EVs-treated AD rats showed noticeably reduced Aβ deposition area, plaques, and Aβ contents (all *p* < 0.01) (Figure 1B, 1C), as well as dramatically decreased soluble Aβ1-42 level in cerebral cortex and hippocampus (all *p* < 0.001) (Figure 1D). Levels of inflammatory cytokines (IL-1β, IL-6, and TNF-α) were also notably decreased after EV treatment (all *p* < 0.001) (Figure 1D). EVs-treated AD rats exhibited markedly elevated NEP and IDE expressions (all *p* < 0.05) (Figure 1E, 1F). From all above, BM-MSC-EVs showed therapeutic effects on AD rats.

### BM-MSC-EVs had therapeutic effects on AD hippocampal neurons

We have verified the therapeutic effects of BM-MSC-EVs on AD rats, and then we further detected *in vitro* effects on hippocampal neurons. The primary hippocampal neurons were firstly cultured. On the 3^rd^ day of culture, the neurons formed neurites, which increased gradually and continued to extend. On the 10^th^ day, the development of neurons became more mature and the neural network became denser (Supplementary Figure 2A); in addition, MAP2 was expressed both in hippocampal neuron body and dendrite under an immunofluorescence microscope (Supplementary Figure 2B).

**Figure 2:**
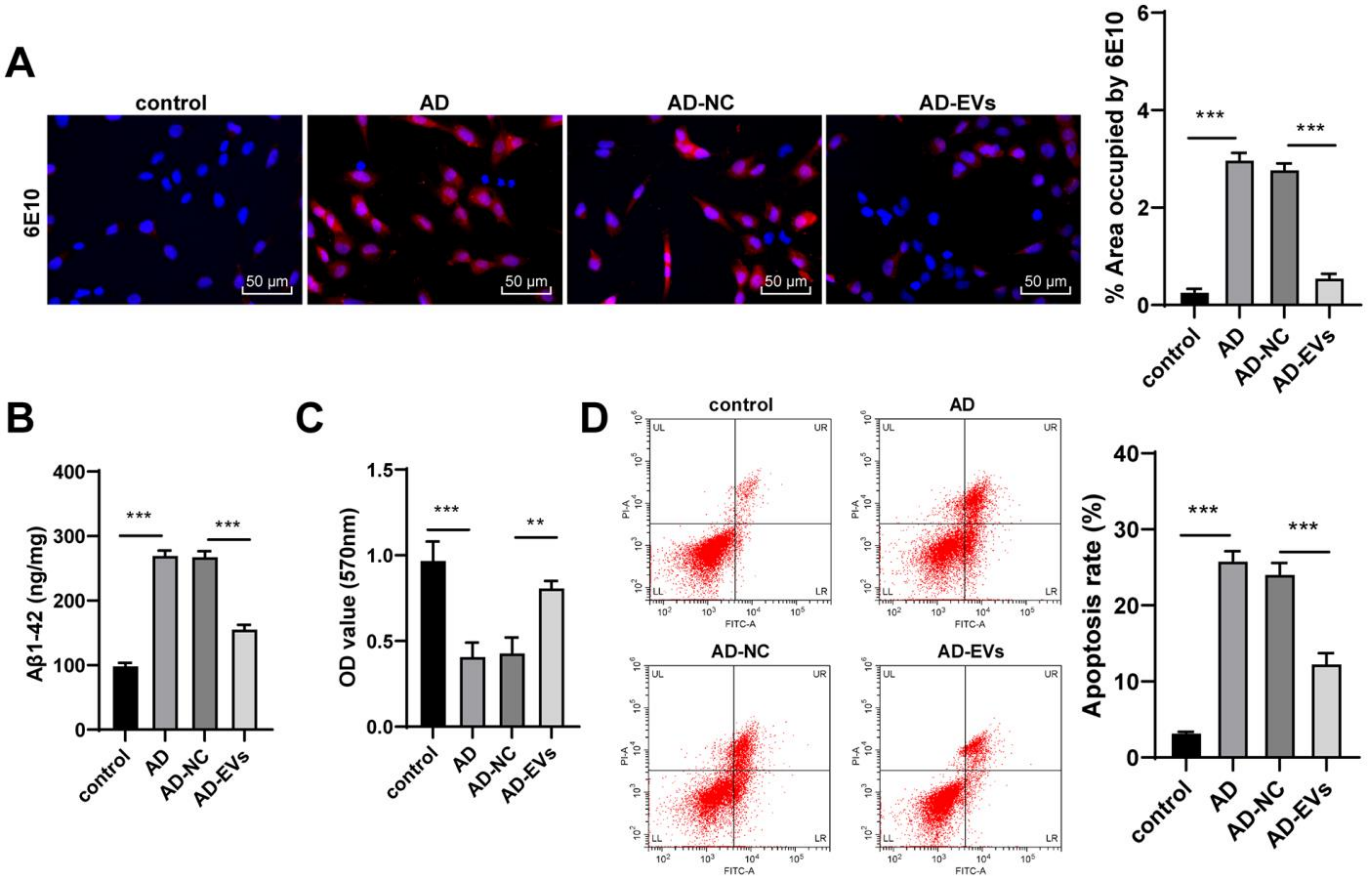
**BM-MSC-EVs have therapeutic effects on AD hippocampal neurons.** The AD neuron model was established by Aβ1-42 induction, and then AD neurons were treated with BM-MSC-EVs or equal volume of BM-MSC conditioned medium after GW4869 treatment. (**A**) Immunofluorescence assay was used to detect Aβ content in hippocampal neurons. (**B**) ELISA was used to detect level of Aβ1-42; (**C**) MTT assay was used to detect the viability of AD hippocampal neurons; (**D**) Flow cytometry was used to detect the apoptosis rate of AD hippocampal neurons. The experiment was repeated three times, and the data was expressed as mean ± standard deviation. Data were analyzed using one-way ANOVA followed by Tukey’s multiple comparisons test. **p < 0.01, ***p < 0.001.

The hippocampal neuron model of AD was established by Aβ1-42. According to immunofluorescence assay and ELISA results, AD neurons showed remarkably increased Aβ deposition area and Aβ plaques, as well as elevated level of Aβ1-42 (all *p* < 0.001) (Figure 2A, 2B). Aβ1-42-challenged neurons also exhibited markedly reduced cell viability and elevated apoptosis rate (all *p* < 0.001) (Figure 2C, 2D). The above observations revealed that the neuron model of AD was successfully established. Next, AD neurons were treated with BM-MSC-EVs. It was found that Aβ deposition area and Aβ plaques were dramatically decreased after EV treatment (both *p* < 0.001) (Figure 2A). EVs-treated AD neurons also exhibited noticeably reduced levels of soluble Aβ1-4 (all *p* < 0.001) (Figure 2B). Additionally, EV treatment obviously increased neuron viability (both *p* < 0.01) (Figure 2C) and reduced apoptosis rate (both *p* < 0.001) (Figure 2D). From all above, we verified that BM-MSC-EVs possessed therapeutic effects on AD hippocampal neurons.

### BM-MSC-EVs carried miR-29c-3p into AD hippocampal neurons

**Figure 3:**
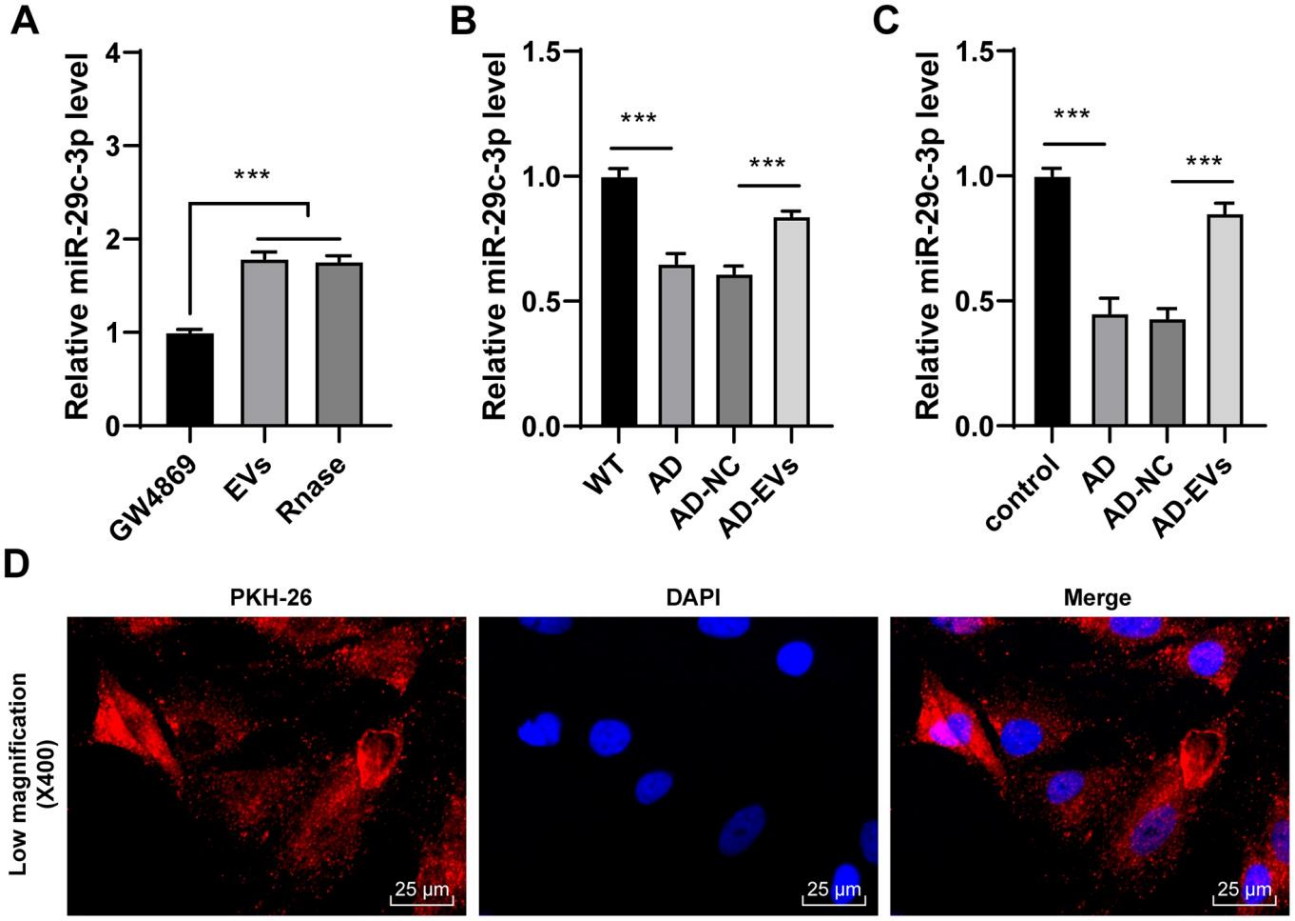
**BM-MSC-EVs carry miR-29c-3p into AD hippocampal neurons.** (**A**) RT-qPCR was used to detect the expression of miR-29c-3p in BM-MSCs and their EVs; (**B**) RT-qPCR was used to detect the expression of miR-29c-3p in cerebral tissues of rats in each group; (**C**) RT-qPCR was used to detect the expression of miR-29c-3p in hippocampal neurons of each group; (**D**) AD neurons treated with PKH-26-labeled EVs were observed under a laser scanning confocal microscopy. The experiment was repeated three times, and the data was expressed as mean ± standard deviation. Data were analyzed using one-way ANOVA followed by Tukey’s multiple comparisons test. ***p < 0.001.

EVs carry a variety of miRs for cellular communication [28–30]. miR-29c-3p is poorly expressed in AD [31]. Hence, we speculated that EVs exerted therapeutic effects on AD by carrying miR-29c-3p. Firstly, miR-29c-3p expression in EVs was detected, and it was found that miR-29c-3p expression was obviously elevated in EVs but showed no difference after the addition of RNase A (*p* < 0.001) (Figure 3A), which confirmed that miR-29c-3p was membrane-encapsulated in EVs. Then miR-29c-3p expression in rats and neurons was detected, as shown by RT-qPCR results, miR-29c-3p was remarkably downregulated in AD rats and neurons, while EV treatment obviously elevated miR-29c-3p expression (all *p* < 0.001) (Figure 3B, 3C). Moreover, AD neurons treated with PKH-26-labeled EVs were observed under a laser scanning confocal microscopy, and we found EVs successfully entered the neurons (Figure 3D). These results indicated that BM-MSC-EVs may be internalized by hippocampal neurons and then release miR-29c-3p to upregulate miR-29c-3p expression in neurons, thus showing therapeutic effects on AD.

### miR-29c-3p knockdown reduced the therapeutic effects of BM-MSC-EVs on AD hippocampal neurons

**Figure 4:**
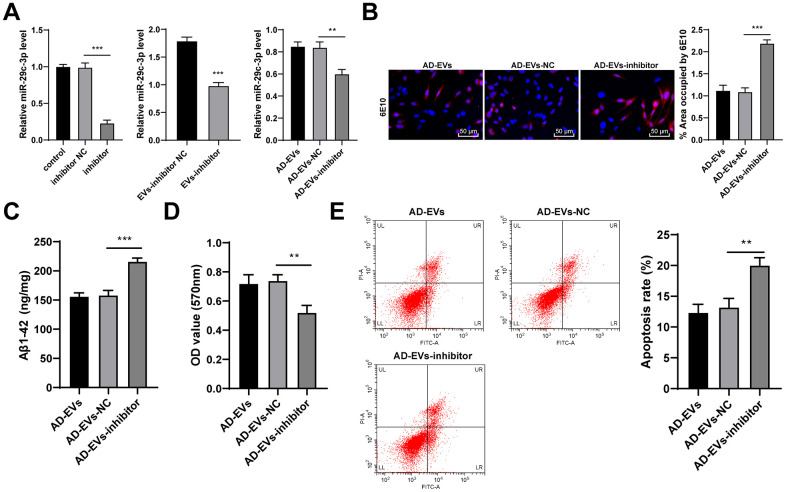
**miR-29c-3p knockdown reduces the therapeutic effects of BM-MSC-EVs on AD hippocampal neurons.** BM-MSCs were transfected with miR-29c-3p inhibitor with inhibitor NC as control. Then EVs were isolated (EVs-inhibitor/ EVs-inhibitor NC) to treat AD neurons. (**A**) RT-qPCR was used to detect the effect of miR-29c-3p inhibitor on the expression of miR-29c-3p in each group; (**B**) Immunofluorescence assay was used to detect the content of Aβ in hippocampal neurons of each group; (**C**) ELISA was used to detect the level of Aβ1-42 in AD hippocampal neurons; (**D**) MTT assay was used to detect the viability of AD hippocampal neurons; (**E**) Flow cytometry was used to detect the apoptosis rate of AD hippocampal neurons. The experiment was repeated three times, and the data was expressed as mean ± standard deviation. Comparisons between two groups were analyzed using independent sample *t*-test; comparisons among multiple groups were analyzed using one-way ANOVA, followed by Tukey’s multiple comparisons test. **p < 0.01; **p < 0.001.

The above mechanism was further verified through experiments. Firstly, miR-29c-3p inhibitor was transfected into BM-MSCs, and the inhibitory effect was verified by RT-qPCR. BM-MSC-EVs were then extracted (EVs-inhibitor) and we observed that miR-29c-3p expression in EVs-inhibitor was notably decreased. Similarly, EVs-inhibitor-treated AD neurons (AD-EVs-inhibitor) also showed obviously reduced miR-29c-3p expression (all *p* < 0.01) (Figure 4A). As shown by immunofluorescence assay results, Aβ deposition area and Aβ plaques in AD-EVs-inhibitor were noticeably increased (*p* < 0.001) (Figure 4B). EVs-inhibitor treatment also markedly upregulated level of Aβ1-42 in AD neurons (all *p* < 0.001) (Figure 4C). Furthermore, according to MTT assay and flow cytometry results, EVs-inhibitor treatment dramatically reduced AD neuron viability (*p* < 0.01) (Figure 4D) and increased apoptosis rate (*p* < 0.01) (Figure 4E). From all above, miR-29c-3p inhibition reduced the therapeutic effects of BM-MSC-EVs on AD hippocampal neurons. We proved that BM-MSC-EVs alleviated AD via carrying and releasing miR-29c-3p into neurons.

### miR-29c-3p targeted BACE1 in AD

**Figure 5:**
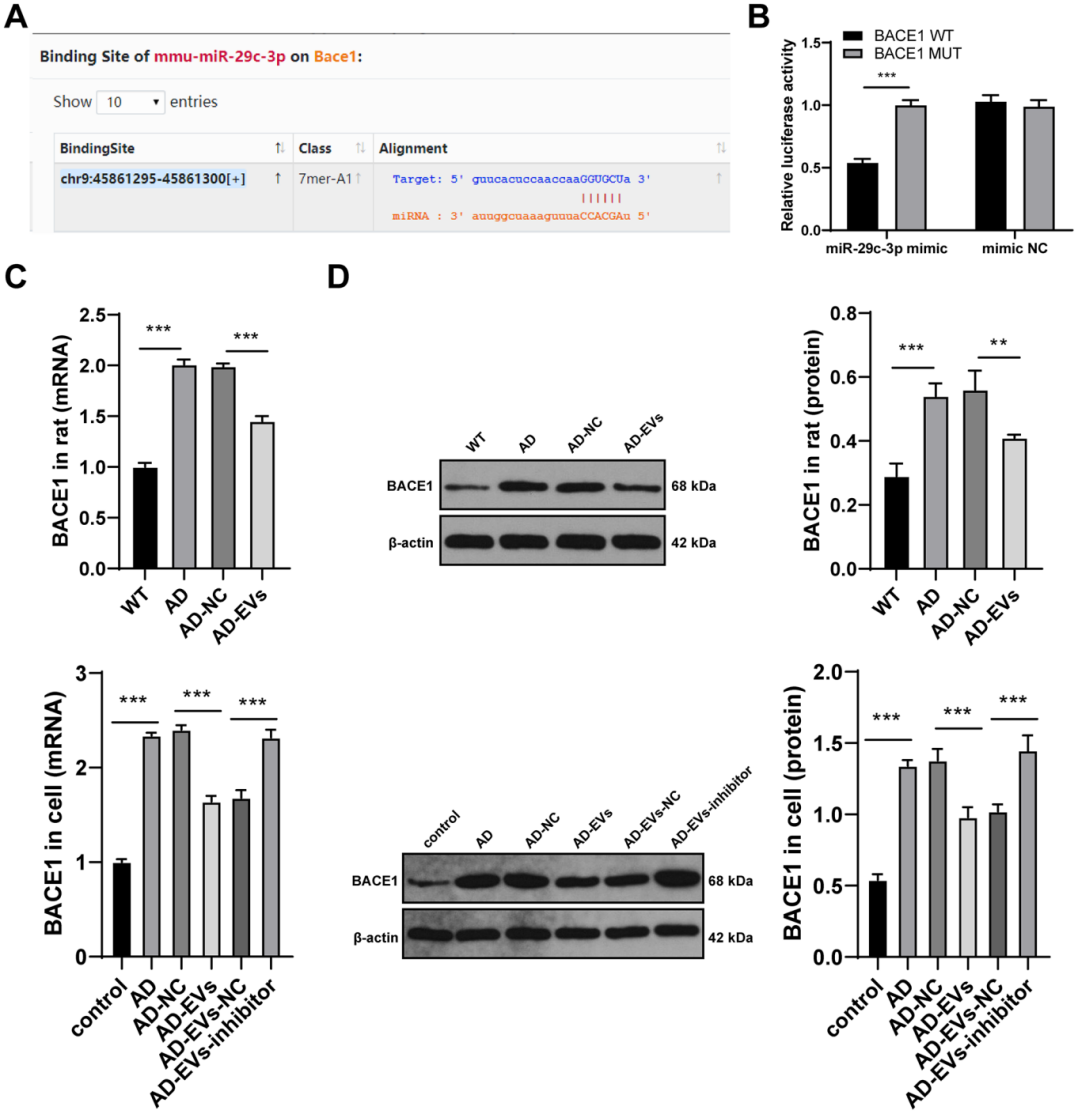
**miR-29c-3p targets BACE1 expression in AD.** (**A**) The binding sites of miR-145-3p and BACE1 were predicted through database (http://starbase.sysu.edu.cn/agoClipRNA.php?source=mRNA); (**B**) Dual-luciferase reporter gene assay was used to verify the binding relationship between miR-145-3p and BACE1; (**C**) RT-qPCR was used to detect the mRNA expression of BACE1 in rat cerebral tissues and neurons in each group; (**D**) WB was used to detect the protein level of BACE1 in rat cerebral tissues and neurons in each group. The experiment was repeated three times, and the data was expressed as mean ± standard deviation. Data were analyzed using one-way ANOVA followed by Sidak’s multiple comparisons test. **p < 0.01; **p < 0.001.

To further reveal the downstream molecular mechanism of EVs-carried miR-29c-3p in AD, we first searched the database (http://starbase.sysu.edu.cn/agoClipRNA.php?source=mRNA) and found that miR-29c-3p has binding sites with multiple genes. Among them, BACE1, an Aβ precursor protein cleaving enzyme, is critical in AD [27, 32, 33]. Hence, we speculated that there may be a binding relationship between miR-29c-3p and BACE1. Based on the binding sites (Figure 5A), the dual-luciferase reporter gene assay verified that miR-29c-3p could target BACE1 (*p* < 0.001) (Figure 5B). Next, BACE1 expression in rat cerebral tissues and hippocampal neurons was detected. As presented by RT-qPCR and WB results, BACE1 expression in AD rats was notably elevated, which was reversed after EVs treatment (both *p* < 0.01) (Figure 5C, 5D). Similar results were also found in hippocampal neurons (both *p* < 0.001) (Figure 5C, 5D). These findings indicated that BACE1 was highly expressed in AD, but EVs treatment inhibited BACE1 expression in AD. Additionally, BACE1 expression in EVs-inhibitor-treated AD neurons was detected and it was found to be upregulated obviously (all *p* < 0.001) (Figure 5C, 5D). Collectively, we confirmed that BM-MSC-EVs-carried miR-29c-3p targeted BACE1 expression in AD neurons.

### BACE1 knockdown reversed the effects of EVs-inhibitor on AD

**Figure 6:**
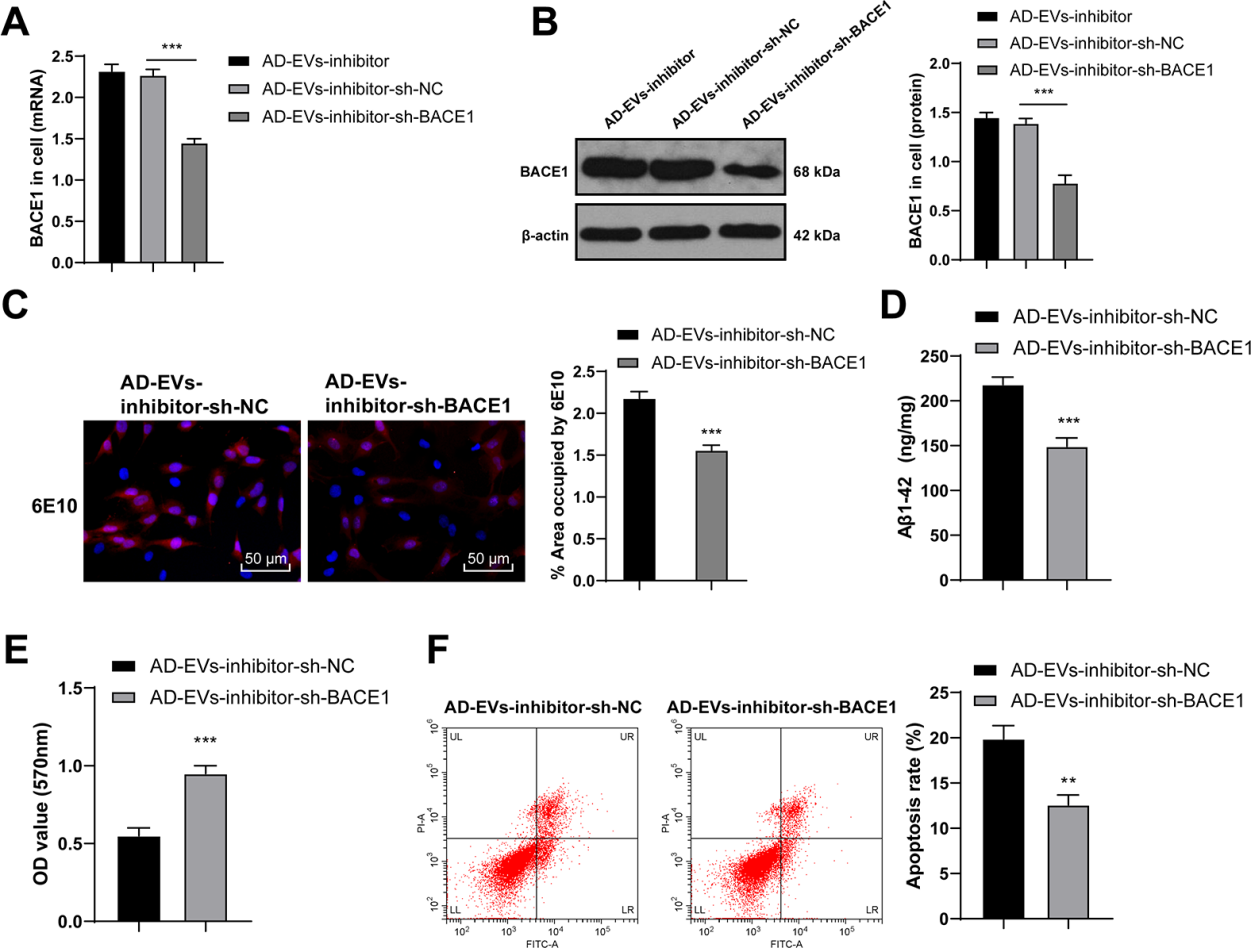
**BACE1 knockdown reverses the effects of EVs-inhibitor on AD.** AD-EVs-inhibitor-treated neurons were transfected with BACE1 shRNA (AD-EVs-inhibitor-sh-BACE1), with AD-EVs-inhibitor-treated neurons transfected with sh-NC (AD-EVs-inhibitor-sh-NC) as control. (**A**, **B**) RT-qPCR and WB were used to detect the silencing effect of shRNA on BACE1; (**C**) Immunofluorescence assay was used to detect the content of Aβ in AD hippocampal neurons; (**D**) ELISA was used to detect level of Aβ1-42; (**E**) MTT assay was used to detect the viability of AD hippocampal neurons; (**F**) Flow cytometry was used to detect the apoptosis rate of AD hippocampal neurons. The experiment was repeated three times, and the data were expressed as mean ± standard deviation. Comparisons between two groups were analyzed using independent sample *t*-test; comparisons among multiple groups were analyzed using one-way ANOVA, followed by Tukey’s multiple comparisons test or Sidak’s multiple comparisons test. ***p < 0.001.

To verify the role of BACE1 in AD, we transfected AD-EVs-inhibitor-treated neurons with BACE1 shRNA (AD-EVs-inhibitor-sh-BACE1), with AD-EVs-inhibitor-treated neurons transfected with sh-NC (AD-EVs-inhibitor-sh-NC) as control. Firstly, RT-qPCR and WB confirmed the successful intervention of sh-BACE1 (both *p* < 0.001) (Figure 6A, 6B). According to our results, Aβ deposition area and Aβ plaques in the AD-EVs-inhibitor-sh-BACE1 group were dramatically reduced relative to those in the AD-EVs-inhibitor-sh-NC group (*p* < 0.001) (Figure 6C). Level of soluble Aβ1-42 was also markedly decreased after BACE1 knockdown in EVs-inhibitor-treated AD neurons (all *p* < 0.001) (Figure 6D). As shown by MTT assay and flow cytometry results, cell viability was markedly increased (*p* < 0.001) (Figure 6E) and apoptosis rate was decreased (*p* < 0.01) (Figure 6F) after silencing BACE1 in EVs-inhibitor-treated neurons. These results suggested that inhibition of BACE1 reversed the effects of EVs-inhibitor on AD hippocampal neurons.

### BM-MSC-EVs activated the Wnt/β-catenin pathway via the miR-29c-3p/BACE1 axis

**Figure 7:**
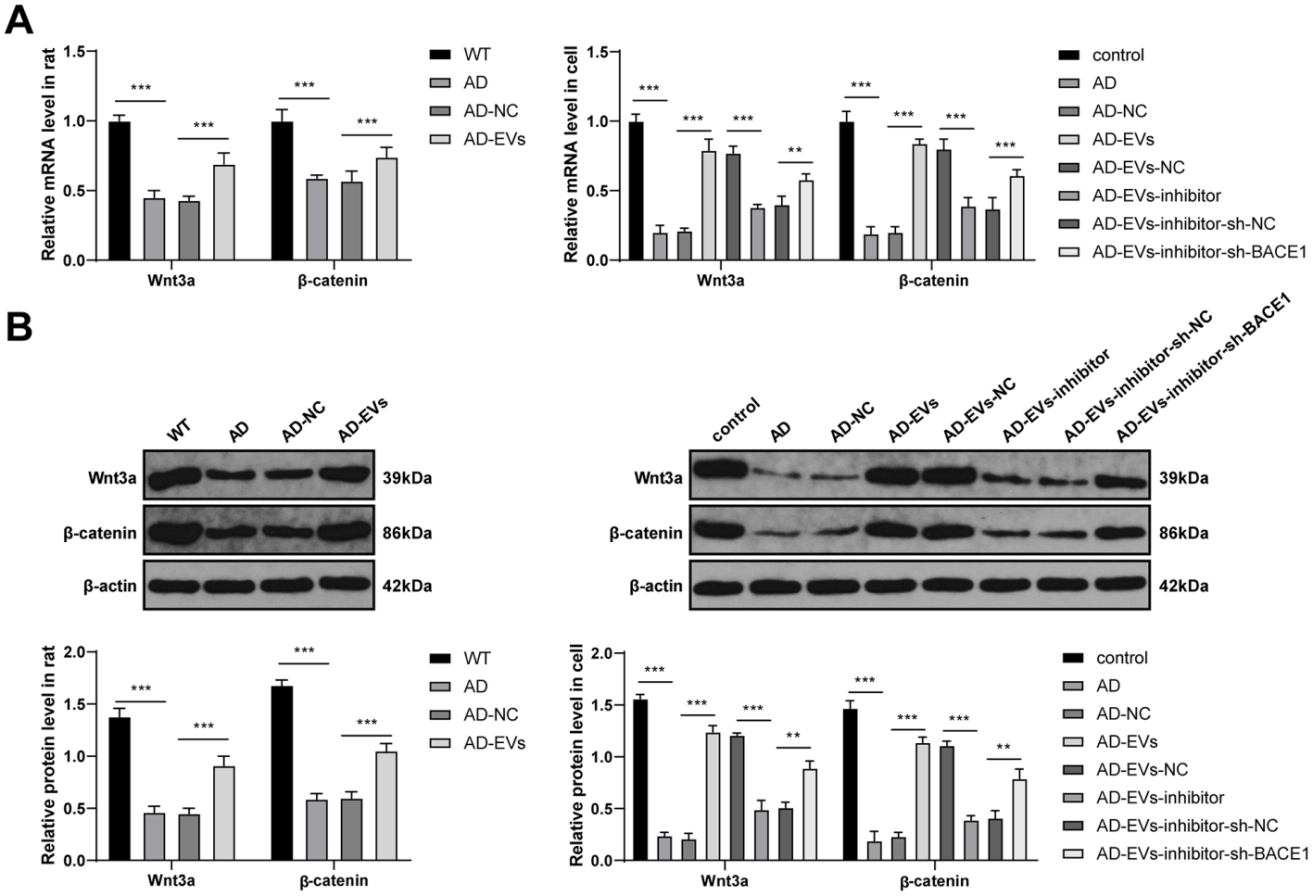
**BM-MSC-EVs activate the Wnt/β-catenin pathway via the miR-29c-3p/BACE1 axis.** (**A**, **B**) RT-qPCR and WB were used to detect the effects of the miR-29c-3p/BACE1 axis on the mRNA expression and protein level of the Wnt/β-catenin pathway-related factors (Wnt3a and β-catenin) during EVs treating AD. The experiment was repeated three times, and the data were expressed as mean ± standard deviation. Data were analyzed using one-way ANOVA followed by Tukey’s multiple comparisons test. **p < 0.01; **p < 0.001.

We have confirmed that BACE1 was the downstream target gene of miR-29c-3p. The downstream molecular mechanism of BACE1 in EV therapeutic effects on AD was further explored. Studies have shown a negative regulatory relationship between BACE1 and the Wnt/β-catenin pathway [34, 35]. The Wnt/β-catenin pathway is inhibited in AD [36, 37]. Therefore, we speculated that the miR-29c-3p/BACE1 axis affected the therapeutic effects of EVs on AD via the Wnt/β-catenin pathway. According to RT-qPCR and WB results, the levels of the Wnt/β-catenin pathway-related factors (Wnt3a and β-catenin) were notably decreased in AD rats and neurons, while they were dramatically increased after EV treatment (all *p* < 0.001) (Figure 7A, 7B). Additionally, after inhibiting miR-29c-3p in BM-MSCs, Wnt3a and β-catenin levels were noticeably reduced in EVs-treated AD neurons (all *p* < 0.001) (Figure 7A, 7B), which were then obviously restored after BACE1 knockdown (all *p* < 0.01) (Figure 7A, 7B). These results suggested that BM-MSC-EVs activated the Wnt/β-catenin pathway via the miR-29c-3p/BACE1 axis.

### Inhibition of the Wnt/β-catenin pathway impaired the therapeutic effects of BM-MSC-EVs on AD

**Figure 8:**
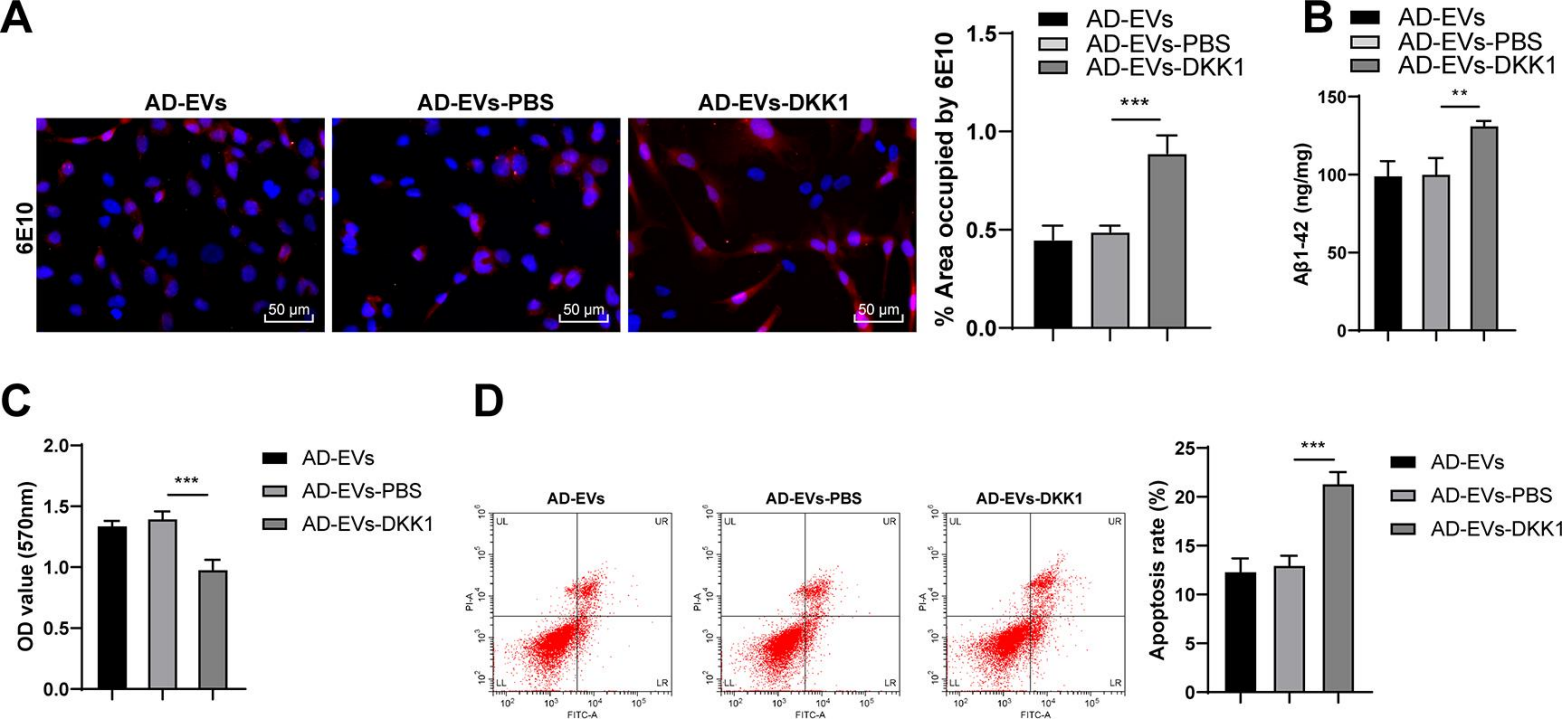
**Inhibition of the Wnt/β-catenin pathway impairs the therapeutic effects of BM-MSC-EVs on AD.** The Wnt/β-catenin pathway inhibitor DKK1 was added to BM-MSC-EVs-treated AD neurons, with the addition of PBS as control. (**A**) The content of Aβ in AD hippocampal neurons was detected using immunofluorescence assay; (**B**) Level of Aβ1-42 in AD hippocampal neurons was detected using ELISA; (**C**) MTT assay was used to detect the viability of AD hippocampal neurons; (**D**) Flow cytometry was used to detect the apoptosis rate of AD hippocampal neurons. The experiment was repeated three times, and the data were expressed as mean ± standard deviation. Data among groups were compared using one-way ANOVA, followed by Tukey’s multiple comparisons test was used. **p < 0.01; ***p < 0.001.

To verify the role of the Wnt/β-catenin pathway in the effects of BM-MSC-EVs-carried miR-29c-3p on AD, we conducted a combined experiment. The Wnt/β-catenin pathway inhibitor DKK1 was added to BM-MSC-EVs-treated AD neurons. As shown by immunofluorescence staining results, Aβ deposition area and Aβ plaques were notably increased after inhibition of the Wnt/β-catenin pathway (*p* < 0.001) (Figure 8A). ELISA results revealed that soluble Aβ1-42 level was also remarkably elevated after silencing the Wnt/β-catenin pathway (all *p* < 0.01) (Figure 8B). Moreover, the cell viability was dramatically decreased (*p* < 0.001) (Figure 8C) and the apoptosis rate was notably increased (*p* < 0.001) (Figure 8D) after blocking the Wnt/β-catenin pathway. Briefly, we proved that inhibition of the Wnt/β-catenin pathway impaired the therapeutic effects of BM-MSC-EVs on AD.

## DISCUSSION

**Figure 9:**
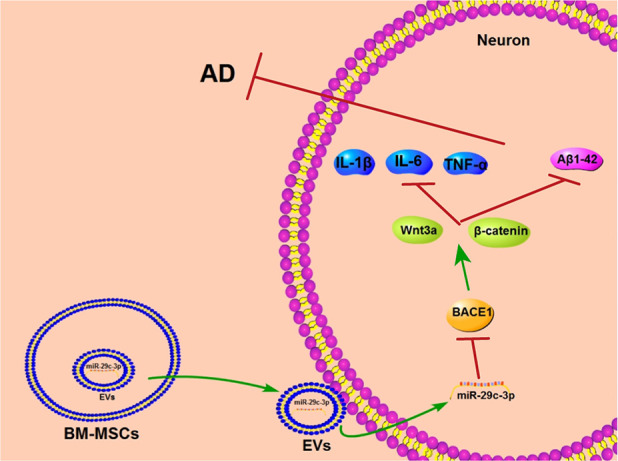
**BM-MSC-EVs-carried miR-29c-3p inhibits BACE1 in AD.** BM-MSC-EVs could be internalized by neuronal cells and then released their carried miR-29c-3p to upregulate miR-29c-3p expression in neurons. The upregulation of miR-29c-3p inhibited BACE1 and then activated the Wnt/β-catenin pathway to reduce levels of Aβ1-42 and inflammatory cytokine (IL-1β, IL-6 and TNF-α), thereby playing a therapeutic role in the treatment of AD.

AD is an irreversible progressive neurodegenerative disease and causes typical cognitive impairment with no effective treatment currently [38, 39]. MSC-EVs show therapeutic potential to alleviate age-related disorders [40]. The current study demonstrated that BM-MSC-EVs ameliorated AD via carrying miR-29c-3p into neurons to target BACE1 and activating the Wnt/β-catenin pathway (Figure 9).

Numerous studies have provided compelling evidence that BM-MSC-EVs are promising candidates for treating multiple neurological diseases with their robust properties of anti-inflammation and neuroprotection [41, 42]. In the current study, following extensive characterizations of EVs isolated from BM-MSCs, we investigated the effects of BM-MSC-EVs on AD. It is well accepted that non-physiological deposition of Aβ leads to AD pathology [43]. High Aβ1-42 level is related to AD genesis and progression [44]. Decreased expressions of NEP and IDE (two critical Aβ-degrading enzymes) result in the promotion of Aβ deposition, thus aggravating AD [45]. According to our results, EV treatment remarkably improved AD rat behaviors, reduced Aβ deposition and plagues, decreased levels of soluble Aβ1-42 and inflammatory cytokines (IL-1β, IL-6, and TNF-α), and elevated NEP and IDE expressions. Similar results were further found in EVs-treated AD neurons, together with increased neuron viability and reduced apoptosis. Consistently, BM-MSC-EVs have been shown to effectively inhibit Aβ plaque formation and carry the enzymatically active NEP, thereby promoting Aβ degrading and then alleviating AD [46]. MSC-EVs possess anti-inflammatory and Aβ-degrading effects, showing protection against neuronal death and memory impairment in AD [47]. From all above, we proved that BM-MSC-EVs showed therapeutic effects on AD rats and neurons.

As has been pointed out previously, EVs are promising biomarker sources for neurodegenerative disease therapy, which is attributable to their transfer of various functional components including miRs in intercellular communication [7]. EVs can carry a variety of miRs for intercellular communication [28–30]. It has been demonstrated that miR-29c-3p is present in MSC-EVs [18]. Importantly, miR-29c-3p is downregulated in AD [31]. However, the mechanism of MSC-EVs-carried miR-29c-3p in AD remained unclear. We detected the expression of miR-29c-3p in BMSC-EVs, and found that BMSC-EVs carried miR-29c-3p, and BMSC-EVs could be internalized by neurons, and upregulate the expression of miR-29c-3p in AD neurons. Therefore, we believed that BMSC-EVs carried miR-29c-3p into neurons. In brief, miR-29c-3p was obviously elevated in EVs, and remarkably downregulated in AD rats and neurons, while EV treatment obviously elevated miR-29c-3p expression in AD. Likewise, a previous work has pointed out that stem cell-derived EVs show therapeutic effects on AD via shuttling their biologically active cargoes including miRs [48]. miR-29c-3p is tightly implicated in AD with its aberrantly downregulated expression, showing the strong potential of being an effective biomarker for AD [17, 49]. Briefly, BM-MSC-EVs carried miR-29c-3p into neurons. Thereafter, we silenced miR-29c-3p expression in BM-MSCs to further verify the effects of EVs on AD. As shown by our results, miR-29c-3p inhibition in BM-MSCs notably reduced the therapeutic effects of BM-MSC-EVs on AD. In support of these, reduced miR-29c-3p expression is intrinsically associated with Aβ-mediated inhibited neuron viability and increased apoptosis in AD [31]. Aberrant miR-29c-3p expression is associated with neuroinflammation in AD [50]. Taken together, BM-MSC-EVs alleviated AD via carrying and releasing miR-29c-3p into neurons.

Next, we shifted to investigating the downstream mechanism of EVs-carried miR-29c-3p in AD. The online website predicted that there are many target genes downstream of miR-29c-3p, among which BACE1 plays a vital in AD [27, 32, 33]. It is well-established that BACE1 induces the initial cleavage of AβPP, greatly contributing to the generation of Aβ and then the promotion of AD pathology [51]. Hence, we chose BACE1 as the target gene to explore the mechanism of miR-29c-3p and BACE1 in AD. Additionally, emerging studies have provided robust evidence that BACE1 exerts effects on AD progression via miR-mediated regulation [52, 53]. We proved that BACE1 was highly expressed in AD and miR-29c-3p targeted BACE1. In line with this, a prior work has also pointed out the targeted regulatory relationship between miR-29c-3p and BACE1 in AD-associated pathologies [50]. Moreover, we found that silencing BACE1 reversed the adverse effects of EV-carried miR-29c-3p knockdown on AD. Likewise, as has been demonstrated, downregulated BACE1 is tightly linked to the decrease of Aβ production and deposition, and eventually to the reduction of inflammation and neuron apoptosis [54]. Briefly, BM-MSC-EVs alleviated AD via the miR-29c-3p/BACE1 axis.

Subsequently, the downstream mechanism of BACE1 was further explored. It has shown that the deregulated Wnt/β-catenin pathway underlies AD onset and progression [55]. Activation of the Wnt/β-catenin signaling suppresses Aβ production and tau protein hyperphosphorylation in the brain, and critically, restoring Wnt/β-catenin signaling represents a unique target for the design of novel AD therapies [56]. According to our observations, the levels of the Wnt/β-catenin pathway-related factors (Wnt3a and β-catenin) were notably decreased in AD rats and neurons, while they were dramatically increased after EV treatment. After inhibiting miR-29c-3p in BM-MSCs, Wnt3a and β-catenin levels were noticeably reduced in EVs-treated AD neurons, which were then obviously restored after BACE1 knockdown. Notably, miR-29c-3p inhibitor and sh-BACE1 treatments could not completely eliminate the effects of miR-29c-3p and BACE1, so the Wnt/β-catenin level in the AD-EVs-inhibitor-sh-BACE1 group did not restore completely. Briefly, this study demonstrated that BM-MSC-EVs-carried miR-29c-3p targeted BACE1 and then activated the Wnt/β-catenin pathway in AD. Accordingly, accumulating studies have highlighted the negative regulatory relationship between BACE1 and the Wnt/β-catenin pathway in AD [34–36]. Moreover, inhibition of the Wnt/β-catenin pathway impaired the therapeutic effects of BM-MSC-EVs on AD. Blockade of the Wnt signaling in AD increases Aβ formation, drives neuroinflammation, and leads to neuronal death [57, 58]. Collectively, we proved that BM-MSC-EVs-carried miR-29c-3p targeted BACE1 and then activated the Wnt/β-catenin pathway, thus ameliorating AD.

All in all, this study supported that BM-MSC-EVs ameliorated AD via the miR-29c-3p/BACE1 axis and then the Wnt/β-catenin pathway activation. These results discovered a novel BM-MSC-EVs-based therapy for AD patients. There were many deficiencies in this study. For example, we didn't buy transgenic animals because of the shortage of research funds, but we reviewed the previous literature and found that intracerebroventricular injection of Aβ can effectively establish the animal model of AD and simulate the pathological state of AD [25, 28, 59, 60]. For these two reasons, we chose to establish a rat model of AD. We will adopt more rigorous transgenic animals to carry out experiments if the research funds permit in the future. Moreover, EVs can carry a variety of miRs for intercellular communication [28–30], and there are many target genes downstream of miR-29c-3p. This study failed to determine whether other miRs or mRNAs played a role in the treatment of AD, and the clinical application of BM-MSC-EVs in AD needs further verification. In the future, we will further explore the functions of other miRs and mRNAs in AD, and determine whether there is a mutual regulatory relationship between the Wnt/β-catenin pathway and BACE1.

## MATERIALS AND METHODS

### Ethics statement

This study was conducted with a firm compliance to the National Institutes of Health Guidelines for the Care and Use of Laboratory Animals, and got approval from the animal Ethics Committee of the First Affiliated Hospital of China Medical University. Significant efforts were made to reduce animal numbers and their pain.

### Isolation and culture of BM-MSCs

BM-MSCs were isolated and cultured using the whole bone marrow adherence method. Sprague Dawley (SD) rats (5-6 weeks; Beijing HFK Biotechnology Co., Ltd., Beijing, China) were anesthetized by intraperitoneal injection of 3% pentobarbital sodium and disinfected with 75% ethanol for 5 min. Under aseptic conditions, bilateral femurs and tibias were separated and metaphysis was opened to expose the medullary cavity. Then the bone marrow was obtained after washing with low glucose-Dulbecco’s Modified Eagle’s Medium (L-DMEM) (Corning Life Sciences, Lowell, MA, USA). Following a centrifugation (100 g, 5 min), the supernatant was discarded and the precipitate was resuspended in L-DMEM containing 10% fetal bovine serum (FBS) (Gibco Company, Grand Island, NY, USA). The cell suspension was seeded (1 × 10^6^ cells/mL) and cultured in an incubator (37° C, 5% CO_2_). After incubation for 24 h, half of the medium was refreshed, and then the medium was refreshed every 2 days. When growing to 80-90% confluence, the cells were passaged at a ratio of 1:2. The morphology of BM-MSCs was observed under an inverted microscope (Zeiss Inc., AG, Oberkochen, Germany). BM-MSCs of the 3^rd^ generation were selected for detection of the expression of stem cell surface markers [(cluster of differentiation (CD)45 (ab33916), CD34 (ab81289), CD29 (ab36219) and CD90 (ab226) (all from Abcam Inc., Cambridge, MA, USA)] using flow cytometry. In addition, other BM-MSCs of the 3^rd^ generation were cultured in osteogenic medium (Sigma-Aldrich, Merck KGaA, Darmstadt, Germany) and adipogenic medium (Cyagen Biosciences, Santa Clara, CA, USA), and stained with alizarin red (Cyagen Biosciences) and Oil red O (Sigma-Aldrich) to analyze the osteogenic and adipogenic differentiation potential correspondingly.

### Isolation and grouping of BM-MSC-EVs

When BM-MSCs of the 3^rd^ generation reached 80% confluence, the cells were rinsed with phosphate-buffered saline (PBS) and the conventional medium was replaced with EVs-free FBS medium (System Biosciences, Mountain View, CA, USA). After a 48-h incubation, the cells were harvested and subjected to several centrifugations (300 g/5 min; 2000 g/10min, 10000 g/35 min). Afterwards, the supernatant was filtered through a 0.22 μm membrane (Merck Millipore Ltd., Tullagreen, Ireland), followed by twice ultracentrifugation (100 000 g, 2 h). The EVs were finally resuspended in 50-100 μL PBS with protein concentration measured using the bicinchoninic acid (BCA) kit (Beyotime Biotechnology Co., Ltd., Shanghai, China), and then stored at -80° C for further experiments. Similarly, BM-MSCs were cultured in EVs-free FBS medium added with GW4869 (20 μg/mL; Sigma-Aldrich) and 48 h later the conditioned medium was taken as control (GW4869 group). According to a previous study [61], the expression of CD9 (ab92726, Abcam), CD63 (ab108950, Abcam) and calnexin (ab22595, Abcam) was detected using Western blotting (WB). EV morphology was observed under a transmission electron microscope (TEM) [26], and the particle size of EVs was detected using a nanoparticle tracking analyzer (NTA) (Malvern Instruments, Ltd., Malvern, UK) [26].

### Animal grouping and treatment

A total of 24 SD rats (200-220 g, 6-7 weeks, female-male ratio of 1:2) were randomly allocated into normal control group [wild-type (WT)], model group (AD), model control group [AD-negative control (NC)] and model treatment group (AD-EVs), 6 rats in each. Rats in the AD group, AD-NC group, and AD-EVs group were injected with oligomer Aβ1-42 (5 μg/μL) (Sigma-Aldrich) stereotaxically from lateral ventricle (anteroposterior: -0.8 mm; mediolateral: -1.4 mm; dorsoventral: -4.0 mm), and the needle was kept for 5 min and then withdrawn slowly. Five days after Aβ1-42 injection, rats in the AD-EVs group were injected with 30 μg EVs (dissolved in 100 μL PBS) at the same position in lateral ventricle; rats in the AD-NC group were injected with the same amount of BM-MSC conditioned medium after GW4869 treatment. The injection was performed once a month at the same time for two months. Behavioral experiments were conducted three weeks after the last injection.

### Morris water maze test

Morris water maze test [22] was used for the evaluation of rat learning and memory abilities. The rats were placed in a circular pool filled with water (1.2 m in diameter, 25 ± 1° C), and visual signals were placed around the pool. The water maze experiment consisted of a visible test (day 0), a hidden platform test (days 1-5), and a probe test (day 6). During the first two tests, rats were put into the pool four times a day, and escape latency (time to find the hidden platform) and swimming speed were recorded. For those rats that did not find the platform within 60 s, they were placed on the platform for 15 s. During the probe test, rats were released and allowed to swim freely for 60 s in a pool without platform, and the percentage of time in each quadrant was recorded.

### Tissue sample collection

After behavioral tests, rats were anesthetized by intraperitoneal injection of pentobarbital sodium (50 mg/kg) and then immediately perfused with PBS (containing 4% paraformaldehyde). Afterwards, the brain was removed on ice and the left and right hemispheres were quickly separated with the cortex and hippocampus of each hemisphere taken out. Part of them was put into the eppendorf tube marked in advance, frozen in liquid nitrogen, and then stored at -80° C for subsequent experiments. Another part was fixed overnight and incubated with 30% sucrose at 4° C until equilibrium. The coronal sections were prepared using a cryostat (CM1850; Leica Microsystems, Wetzlar, Germany) and preserved at 4° C.

### Thioflavin S staining

The free-floating sections (30 μm) were subjected to a 10-min incubation with 1% Thioflavin S (Beijing Solarbio Science and Technology Co., Ltd., Beijing, China) dissolved in 50% ethanol, 2 washes with 50% ethanol, 5 min each, and then a 5-min washing with distilled water. Next, the sections were mounted using Vectashield fluorescent mounting medium (H-1000, Shanghai Sanger Biotechnology Co., Ltd., Shanghai, China). Under the fluorescence microscope (Olympus Optical Co., Ltd., Tokyo, Japan), the senile plaques were shown as green spots. Statistical analysis was performed using ImageJ 1.48 (National Institutes of Health, Bethesda, MD, USA).

### Extraction and identification of hippocampal neurons

SD rats after birth within 24 h were washed with 75% alcohol, transferred into the intercellular space and placed in 75% ice ethanol for 2-3 min. The rats were sacrificed by decapitation and their cerebral tissues were rapidly stripped. Following 3 washes with precooled D-Hanks (Solarbio), the hippocampus was dissected from the brain under a stereomicroscope (Olympus), and washed three times with PBS to make the tissues sink to the bottom of the test tube. Afterwards, the hippocampus underwent a 30-min detachment in a water bath at 30° C using 2 mg/mL papain solution (in Hibernate A), and a 12-min detachment using 20-60 μL DNase I (5 units/μL) (Thermo Fisher Scientific Inc., Waltham, MA, USA). Hippocampus that failed to be detached was filtered using a wet cell filter (40-mm mesh, BD Biosciences, San Jose, CA, USA) and centrifuged (1806 g, 10 min). Next, the particles were resuspended in a plating media with 15% heat-inactivated horse serum (HI-HS), 5% HI-FBS, 2% B27, 0.5 mM glutamine, 1% N21-MAX and 0.5% P/S + Genta. + Amph. Neurobasal A was supplemented with B and placed on a dish precoated with poly-D-lysine (10 μg). At 2-4 h post-incubation, the medium containing 3% HI-HS, 2% B27, 0.5 mM glutamine, 1% N21-MAX and 0.5% P/S + Genta. + Amph was used. Half of the culture medium was replaced with fresh serum-free neurobasal A every day. The culture was added with 2.5-5 μM cytosine arabinoside on day 3-4 to inhibit glial cell growth, and half of the medium was refreshed every 2 days. The morphology and differentiation of neurons were evaluated [62, 63]. Neurons were identified using immunofluorescence.

### PKH-26-labeled BM-MSC-EVs

EVs were labeled with PKH-26 (Sigma-Aldrich) as per the manufacturer’s instructions. In short, EVs particles (75 μg) were resuspended in 1 mL diluent C. In addition, 2 μL PKH-26 was mixed with 245 μL diluent C. EVs suspension was added to staining solution for a 5-min incubation, which was terminated by the addition of 1% bovine serum albumin (BSA). Afterwards, the neurons were seeded on 24-well slides and PKH-26-labeled EVs (10 μg) were added into the medium. Following the fixing with 4% formaldehyde for 4 h, 4’,6-diamidino-2-phenylindole (Sigma-Aldrich) was used for nucleus staining. Finally, the hippocampal neurons were observed for EV internalizations using a laser scanning confocal microscopy (LSM710, Zeiss).

### Hippocampal neuron treatment and grouping

The cell model of AD was induced by a 24-h treatment of 10 μmol/L Aβ1-42 (Sigma-Aldrich). Neurons were allocated into control, AD, AD-NC (AD neurons treated with an equal volume of BM-MSC conditioned medium after GW4869 treatment) and AD-EVs (AD neurons treated with 30 μg EVs dissolved in 100 μL PBS) groups. The subsequent experiments were conducted after 24 h.

miR-29c-3p inhibitor and its NC (100 pmol; Shanghai GenePharma Co., Ltd., Shanghai, China) were transfected into BM-MSCs using Lipofectamine 2000 (Thermo Fisher). Following a 4-h incubation at 37° C, the BM-MSCs were further cultured in the complete medium for 48 h. After the transfection efficiency was determined using reverse transcription quantitative polymerase chain reaction (RT-qPCR), EVs were isolated and used to treat AD neurons (AD-EVs-NC and AD-EVs-inhibitor groups). After 24 h, further experiments were carried out.

AD neurons were treated with short hairpin (sh)-beta-site amyloid precursor protein-cleaving enzyme 1 (BACE1) (AD-EVs-inhibitor-sh-BACE1 group) and sh-NC (AD-EVs-inhibitor-sh-NC group) (1.0 × 10^8^ TU/mL, GenePharma). The further experiments were carried out 24 h later.

AD neurons were treated with a Wnt/β-catenin pathway inhibitor Dickkopf-1 (DKK1) (200 ng/mL; PeproTech, Rocky Hill, NJ, USA) as per the manufacturer’s instructions. The AD neurons were allocated into AD-EVs-PBS and AD-EVs-DKK1 groups. The neurons were collected 48 h later for further experiments.

### Immunofluorescence assay

Immunofluorescence assay was performed as previously described [64]. The antibodies used were anti-microtubule-associated protein 2 (MAP2) (ab32454, Abcam) and anti-6E10 (803001, BioLegend, San Diego, CA, USA). To facilitate observation, the Alexa-Fluor488-conjugated (ab150077, Abcam) or Alexa-Fluor594-conjugated (ab150116, Abcam) secondary antibody was used for a 1-h incubation at room temperature. BX51 immunofluorescence microscope (Olympus) was used for observation.

### 3-(4,5-Dimethylthiazol-2-yl)-2,5-diphenyltetrazolium bromide (MTT) assay

AD neuron viability was detected using MTT assay [65]. AD hippocampal neurons (5×10^5^ cells/mL) were seeded into 96-well plates with 5 × 10^5^/mL, and intervened according to the experimental groups. Following a 24-h incubation with 5% CO_2_, 10 μL MTT (5 mg/mL in PBS) was added into each well for a 4-h incubation at 37° C. Afterwards, the culture medium was removed, and each well was added with 100 μL dimethyl sulfoxide. After the particle dissolution, the optical density (OD) at 570 nm of each group was measured using a microplate reader (Power Wave XS, BioTek Instruments Inc., Winooski, VT, USA).

### Flow cytometry

The cells were collected, washed twice with PBS, and resuspended to 1 × 10^6^ cells/L. Cell solution (100 μL) was added with 5 μL fluorescein isothiocyanate-labeled Annexin V and 10 μL propidium iodide, and the mixture was then placed at room temperature away from light for 15 min. Following the addition of 400 μL binding buffer to each tube, a flow cytometer (Beckman Coulter, Inc., Brea, CA, USA) was used for detection within 1 h. The excitation wavelength was 488 nm and 10^4^ cells were counted. All data were collected and processed using CellQuest Pro software (BD).

### Enzyme-linked immunosorbent assay (ELISA)

The left half of the cerebral cortex or hippocampus of each rat was homogenized in a buffer containing 50 mM Tris and 5 M guanidine hydrochloride (pH = 8.0). The homogenate was mixed at room temperature for 4 h and diluted in PBS containing 5% BSA, 0.03% Tween 20 and protease inhibitor cocktail [27, 66, 67]. Aβ1-42 level was detected using a fluorescent kit (Invitrogen Inc., Carlsbad, CA, USA), and protein levels of inflammatory cytokines [interleukin (IL)-1β, IL-6 and tumor necrosis factor-α (TNF-α) in rat cerebral tissues were analyzed using ELISA kits (ZCIBIO Co., Ltd., Shanghai, China). The concentration of each factor was determined by comparing the relative OD of the sample to that of the standard.

### Dual-luciferase reporter gene assay

According to the prediction results of Starbase database (http://starbase.sysu.edu.cn/agoClipRNA.php?source=mRNA) [68], there were target binding sites between BACE1 and miR-29c-3p. Based on these binding sites, WT and mutant (MUT) vectors (Ambion, Austin, TX, USA) of BACE1 were constructed. Next, WT or MUT vectors (0.5 μg) were co-transfected with 40 nmol miR-29c-3p mimic or 40 nmol mimic NC into 293T cells using Lipofectamine 2000 (Thermo Fisher). Cells were harvested at 48 h post-transfection using passive lysis buffer (Ambion) according to the manufacturer’s instructions. The GloMax^®^20/20 luminometer (Promega Corp., Madison, WI, USA) was employed for luciferase activity detection.

### RT-qPCR

Rat cerebral tissues, cell lysates, or EVs were collected. Total RNA was extracted using TRIzol or TRIzol LS (Invitrogen). RNA concentration was determined utilizing the Nanodrop ND-1000 spectrophotometer (Thermo Fisher), and RNA purity was measured by the ratio of A260/A280 (1.8-2.0). The cDNA was synthesized using cDNA synthesis kit (Takara Bio Inc., Otsu, Japan). SYBR Green reagent (BioFact, Daejeon, South Korea) was used for miRs and mRNAs real-time PCR detection. U6 or glyceraldehyde-3-phosphate dehydrogenase (GAPDH) served as the internal reference. Quantitative expression was calculated using the 2^-ΔΔ CT^ method. The experiment was done three times repeatedly. The primers were synthesized by Sangon Biotech Co., Ltd. (Shanghai, China) (Table 1).

**Table 1:** Primer sequences for qRT-PCR.

**Primer**	**Sequences (5’-3’)**	**Accession**
BACE1	F: ATGGCCCCGGCGCTGCGCTGGCTC	NM_019204
	R: TTATTTCAGCAGGGAGATGTCAT	
NEP	F: ATGGGAAGATCAGAAAGTCAGAT	NM_001289462
	R: TCACCAAACCCGACATTTCCTTT	
IDE	F: ATGCGGAACGGGCTCGTGTGGCT	NM_013159
	R: TCAGAGTTTTGCCGCCATGAAGTT	
miR-29c-3p	F: TAGCACCATTTGAAATCGGTTA	MIMAT0000803
	R: TAACCGATTTCAAATGGTGCTA	
Wnt3a	F: ATGTACAACCTCTGTGTGGTGGTG	NM_001107005
	R: CTACTTGCAGGTGTGCACGTCATA	
β-catenin	F: ATGGCTACTCAAGCTGACCTCATG	NM_053357
	R: TTACAGGTCGGTATCAAACCAGGC	
U6	F: ATGGCGGACGACGTAGATCAGCA	NM_001106611
	R: TCAGCCAACTCTCAATGGAGGGGC	
GAPDH	F: ATGGTGAAGGTCGGTGTGAACGGA	NM_017008
	R: TTACTCCTTGGAGGCCATGTAGGC	

### WB

Rat cerebral tissues or cell lysates of the above groups were placed in ice-precooled RIPA lysis buffer (Beyotime) for 30 min, followed by centrifugation (12000 g, 10 min, 4° C). The supernatant was used for analysis. Protein concentration was determined using the BCA (Beyotime) method. Proteins were subjected to 10% sodium dodecyl sulfate-polyacrylamide gel electrophoresis (Beyotime) for separation, transferred to the nitrocellulose membranes (Whatman Inc., Piscataway, NJ, USA), and then blocked for 1 h with blocking solution (P0023B, Beyotime) at room temperature. Afterwards, the membranes underwent an overnight incubation with primary antibodies at 4° C, followed by membrane washing and then a 2-h incubation at room temperature with secondary antibody horseradish peroxidase-labeled immunoglobulin G (IgG) H&L (ab205718, 1:5000, Abcam). The enhanced chemiluminescence working solution (EMD Millipore Corporation, Billerica, MA, USA) was used for development. ImageJ 1.48 (National Institutes of Health) was used for data analysis with β-actin as an internal reference. The primary antibodies (all from Abcam) were neprilysin (NEP) (ab216341, 1:1000), insulin-degrading enzyme (IDE) (ab133561, 1:1000), BACE1 (ab183612, 1:1000), Wnt3a (ab219412, 1:1000), β-catenin (ab32572, 1:5000) and β-actin (ab8227, 1:1000).

### Statistical analysis

All data were processed using SPSS 21.0 (IBM Corp., Armonk, NY, USA). Data were first verified to show normal distribution and homogeneity of variance. Data were expressed in the form of mean ± standard deviation. The independent sample *t*-test was used for comparison analysis between two groups and one-way analysis of variance (ANOVA) was used for comparison among multiple groups followed by Sidak’s multiple Comparisons test or Tukey’s multiple comparisons test. *p* < 0.05 indicated the statistically significant difference.

## Supplementary Material

Supplementary Figures

## References

[1] DeTure MA, Dickson DW. The neuropathological diagnosis of Alzheimer’s disease. Mol Neurodegener. 2019; 14:32. 10.1186/s13024-019-0333-5 31375134 PMC6679484

[2] Nikolac Perkovic M, Pivac N. Genetic Markers of Alzheimer’s Disease. Adv Exp Med Biol. 2019; 1192:27–52. 10.1007/978-981-32-9721-0_3 31705489

[3] Lashley T, Schott JM, Weston P, Murray CE, Wellington H, Keshavan A, Foti SC, Foiani M, Toombs J, Rohrer JD, Heslegrave A, Zetterberg H. Molecular biomarkers of Alzheimer’s disease: progress and prospects. Dis Model Mech. 2018; 11:dmm031781. 10.1242/dmm.031781 29739861 PMC5992610

[4] Forloni G, Balducci C. Alzheimer’s Disease, Oligomers, and Inflammation. J Alzheimers Dis. 2018; 62:1261–76. 10.3233/JAD-170819 29562537 PMC5869993

[5] Guest FL, Rahmoune H, Guest PC. Early Diagnosis and Targeted Treatment Strategy for Improved Therapeutic Outcomes in Alzheimer’s Disease. Adv Exp Med Biol. 2020; 1260:175–91. 10.1007/978-3-030-42667-5_8 32304035

[6] Wong KH, Riaz MK, Xie Y, Zhang X, Liu Q, Chen H, Bian Z, Chen X, Lu A, Yang Z. Review of Current Strategies for Delivering Alzheimer’s Disease Drugs across the Blood-Brain Barrier. Int J Mol Sci. 2019; 20:381. 10.3390/ijms20020381 30658419 PMC6358942

[7] Izadpanah M, Seddigh A, Ebrahimi Barough S, Fazeli SA, Ai J. Potential of Extracellular Vesicles in Neurodegenerative Diseases: Diagnostic and Therapeutic Indications. J Mol Neurosci. 2018; 66:172–79. 10.1007/s12031-018-1135-x 30140997

[8] Vinaiphat A, Sze SK. Clinical implications of extracellular vesicles in neurodegenerative diseases. Expert Rev Mol Diagn. 2019; 19:813–24. 10.1080/14737159.2019.1657407 31429341

[9] Koniusz S, Andrzejewska A, Muraca M, Srivastava AK, Janowski M, Lukomska B. Extracellular Vesicles in Physiology, Pathology, and Therapy of the Immune and Central Nervous System, with Focus on Extracellular Vesicles Derived from Mesenchymal Stem Cells as Therapeutic Tools. Front Cell Neurosci. 2016; 10:109. 10.3389/fncel.2016.00109 27199663 PMC4852177

[10] Szatanek R, Baj-Krzyworzeka M, Zimoch J, Lekka M, Siedlar M, Baran J. The Methods of Choice for Extracellular Vesicles (EVs) Characterization. Int J Mol Sci. 2017; 18:1153. 10.3390/ijms18061153 28555055 PMC5485977

[11] Liew LC, Katsuda T, Gailhouste L, Nakagama H, Ochiya T. Mesenchymal stem cell-derived extracellular vesicles: a glimmer of hope in treating Alzheimer’s disease. Int Immunol. 2017; 29:11–19. 10.1093/intimm/dxx002 28184439

[12] Wang SS, Jia J, Wang Z. Mesenchymal Stem Cell-Derived Extracellular Vesicles Suppresses iNOS Expression and Ameliorates Neural Impairment in Alzheimer’s Disease Mice. J Alzheimers Dis. 2018; 61:1005–13. 10.3233/JAD-170848 29254100

[13] d’Angelo M, Cimini A, Castelli V. Insights into the Effects of Mesenchymal Stem Cell-Derived Secretome in Parkinson’s Disease. Int J Mol Sci. 2020; 21:5241. 10.3390/ijms21155241 32718092 PMC7432166

[14] Qiu G, Zheng G, Ge M, Wang J, Huang R, Shu Q, Xu J. Functional proteins of mesenchymal stem cell-derived extracellular vesicles. Stem Cell Res Ther. 2019; 10:359. 10.1186/s13287-019-1484-6 31779700 PMC6883709

[15] Qiu G, Zheng G, Ge M, Wang J, Huang R, Shu Q, Xu J. Mesenchymal stem cell-derived extracellular vesicles affect disease outcomes via transfer of microRNAs. Stem Cell Res Ther. 2018; 9:320. 10.1186/s13287-018-1069-9 30463593 PMC6249826

[16] Iranifar E, Seresht BM, Momeni F, Fadaei E, Mehr MH, Ebrahimi Z, Rahmati M, Kharazinejad E, Mirzaei H. Exosomes and microRNAs: New potential therapeutic candidates in Alzheimer disease therapy. J Cell Physiol. 2019; 234:2296–305. 10.1002/jcp.27214 30191975

[17] Wu Y, Xu J, Xu J, Cheng J, Jiao D, Zhou C, Dai Y, Chen Q. Lower Serum Levels of miR-29c-3p and miR-19b-3p as Biomarkers for Alzheimer’s Disease. Tohoku J Exp Med. 2017; 242:129–36. 10.1620/tjem.242.129 28626163

[18] Nazari-Shafti TZ, Neuber S, Duran AG, Exarchos V, Beez CM, Meyborg H, Krüger K, Wolint P, Buschmann J, Böni R, Seifert M, Falk V, Emmert MY. MiRNA Profiles of Extracellular Vesicles Secreted by Mesenchymal Stromal Cells-Can They Predict Potential Off-Target Effects? Biomolecules. 2020; 10:1353. 10.3390/biom10091353 32971982 PMC7565205

[19] He D, Xu Y, Xiong X, Yin C, Lei S, Cheng X. The bone marrow-derived mesenchymal stem cells (BMSCs) alleviate diabetic peripheral neuropathy induced by STZ via activating GSK-3β/β-catenin signaling pathway. Environ Toxicol Pharmacol. 2020; 79:103432. 10.1016/j.etap.2020.103432 32502517

[20] Kim J, Lee Y, Lee S, Kim K, Song M, Lee J. Mesenchymal Stem Cell Therapy and Alzheimer’s Disease: Current Status and Future Perspectives. J Alzheimers Dis. 2020; 77:1–14. 10.3233/JAD-200219 32741816

[21] Lee JK, Jin HK, Endo S, Schuchman EH, Carter JE, Bae JS. Intracerebral transplantation of bone marrow-derived mesenchymal stem cells reduces amyloid-beta deposition and rescues memory deficits in Alzheimer’s disease mice by modulation of immune responses. Stem Cells. 2010; 28:329–43. 10.1002/stem.277 20014009

[22] Nakano M, Kubota K, Kobayashi E, Chikenji TS, Saito Y, Konari N, Fujimiya M. Bone marrow-derived mesenchymal stem cells improve cognitive impairment in an Alzheimer’s disease model by increasing the expression of microRNA-146a in hippocampus. Sci Rep. 2020; 10:10772. 10.1038/s41598-020-67460-1 32612165 PMC7330036

[23] Burgio S, Noori L, Marino Gammazza A, Campanella C, Logozzi M, Fais S, Bucchieri F, Cappello F, Caruso Bavisotto C. Extracellular Vesicles-Based Drug Delivery Systems: A New Challenge and the Exemplum of Malignant Pleural Mesothelioma. Int J Mol Sci. 2020; 21:5432. 10.3390/ijms21155432 32751556 PMC7432055

[24] Oszvald Á, Szvicsek Z, Pápai M, Kelemen A, Varga Z, Tölgyes T, Dede K, Bursics A, Buzás EI, Wiener Z. Fibroblast-Derived Extracellular Vesicles Induce Colorectal Cancer Progression by Transmitting Amphiregulin. Front Cell Dev Biol. 2020; 8:558. 10.3389/fcell.2020.00558 32775326 PMC7381355

[25] Nakano M, Nagaishi K, Konari N, Saito Y, Chikenji T, Mizue Y, Fujimiya M. Bone marrow-derived mesenchymal stem cells improve diabetes-induced cognitive impairment by exosome transfer into damaged neurons and astrocytes. Sci Rep. 2016; 6:24805. 10.1038/srep24805 27102354 PMC4840335

[26] Ying H, Lin F, Ding R, Wang W, Hong W. Extracellular vesicles carrying miR-193a derived from mesenchymal stem cells impede cell proliferation, migration and invasion of colon cancer by downregulating FAK. Exp Cell Res. 2020; 394:112144. 10.1016/j.yexcr.2020.112144 32540398

[27] Do J, Kim N, Jeon SH, Gee MS, Ju YJ, Kim JH, Oh MS, Lee JK. Trans-Cinnamaldehyde Alleviates Amyloid-Beta Pathogenesis via the SIRT1-PGC1α-PPARγ Pathway in 5XFAD Transgenic Mice. Int J Mol Sci. 2020; 21:4492. 10.3390/ijms21124492 32599846 PMC7352815

[28] Jahangard Y, Monfared H, Moradi A, Zare M, Mirnajafi-Zadeh J, Mowla SJ. Therapeutic Effects of Transplanted Exosomes Containing miR-29b to a Rat Model of Alzheimer’s Disease. Front Neurosci. 2020; 14:564. 10.3389/fnins.2020.00564 32625049 PMC7314926

[29] Jeppesen DK, Fenix AM, Franklin JL, Higginbotham JN, Zhang Q, Zimmerman LJ, Liebler DC, Ping J, Liu Q, Evans R, Fissell WH, Patton JG, Rome LH, et al. Reassessment of Exosome Composition. Cell. 2019; 177:428–445.e18. 10.1016/j.cell.2019.02.029 30951670 PMC6664447

[30] Minciacchi VR, Freeman MR, Di Vizio D. Extracellular vesicles in cancer: exosomes, microvesicles and the emerging role of large oncosomes. Semin Cell Dev Biol. 2015; 40:41–51. 10.1016/j.semcdb.2015.02.010 25721812 PMC4747631

[31] Liu Z, Zhang H, Sun L, Zhu K, Lang W. miR-29c-3p Increases Cell Viability and Suppresses Apoptosis by Regulating the TNFAIP1/NF-κB Signaling Pathway via TNFAIP1 in Aβ-Treated Neuroblastoma Cells. Neurochem Res. 2020; 45:2375–84. 10.1007/s11064-020-03096-x 32712875

[32] Hernández-Rodríguez M, Arciniega-Martínez IM, García-Marín ID, Correa-Basurto J, Rosales-Hernández MC. Chronic Administration of Scopolamine Increased GSK3βP9, Beta Secretase, Amyloid Beta, and Oxidative Stress in the Hippocampus of Wistar Rats. Mol Neurobiol. 2020; 57:3979–88. 10.1007/s12035-020-02009-x 32638218

[33] Wan L, Zhang Q, Luo H, Xu Z, Huang S, Yang F, Liu Y, Mahaman YA, Ke D, Wang Q, Liu R, Wang JZ, Shu X, Wang X. Codonopsis pilosula polysaccharide attenuates Aβ toxicity and cognitive defects in APP/PS1 mice. Aging (Albany NY). 2020; 12:13422–36. 10.18632/aging.103445 32652518 PMC7377903

[34] Parr C, Mirzaei N, Christian M, Sastre M. Activation of the Wnt/β-catenin pathway represses the transcription of the β-amyloid precursor protein cleaving enzyme (BACE1) via binding of T-cell factor-4 to BACE1 promoter. FASEB J. 2015; 29:623–35. 10.1096/fj.14-253211 25384422

[35] Wang CY, Zheng W, Wang T, Xie JW, Wang SL, Zhao BL, Teng WP, Wang ZY. Huperzine A activates Wnt/β-catenin signaling and enhances the nonamyloidogenic pathway in an Alzheimer transgenic mouse model. Neuropsychopharmacology. 2011; 36:1073–89. 10.1038/npp.2010.245 21289607 PMC3077275

[36] Jin N, Zhu H, Liang X, Huang W, Xie Q, Xiao P, Ni J, Liu Q. Sodium selenate activated Wnt/β-catenin signaling and repressed amyloid-β formation in a triple transgenic mouse model of Alzheimer’s disease. Exp Neurol. 2017; 297:36–49. 10.1016/j.expneurol.2017.07.006 28711506

[37] Zhang X, Yin WK, Shi XD, Li Y. Curcumin activates Wnt/β-catenin signaling pathway through inhibiting the activity of GSK-3β in APPswe transfected SY5Y cells. Eur J Pharm Sci. 2011; 42:540–46. 10.1016/j.ejps.2011.02.009 21352912

[38] Villain N, Dubois B. Alzheimer’s Disease Including Focal Presentations. Semin Neurol. 2019; 39:213–26. 10.1055/s-0039-1681041 30925614

[39] Wang Z, Peng W, Zhang C, Sheng C, Huang W, Wang Y, Fan R. Effects of stem cell transplantation on cognitive decline in animal models of Alzheimer’s disease: A systematic review and meta-analysis. Sci Rep. 2015; 5:12134. 10.1038/srep12134 26159750 PMC4498325

[40] Boulestreau J, Maumus M, Rozier P, Jorgensen C, Noël D. Mesenchymal Stem Cell Derived Extracellular Vesicles in Aging. Front Cell Dev Biol. 2020; 8:107. 10.3389/fcell.2020.00107 32154253 PMC7047768

[41] Doeppner TR, Herz J, Görgens A, Schlechter J, Ludwig AK, Radtke S, de Miroschedji K, Horn PA, Giebel B, Hermann DM. Extracellular Vesicles Improve Post-Stroke Neuroregeneration and Prevent Postischemic Immunosuppression. Stem Cells Transl Med. 2015; 4:1131–43. 10.5966/sctm.2015-0078 26339036 PMC4572905

[42] Kodali M, Castro OW, Kim DK, Thomas A, Shuai B, Attaluri S, Upadhya R, Gitai D, Madhu LN, Prockop DJ, Shetty AK. Intranasally Administered Human MSC-Derived Extracellular Vesicles Pervasively Incorporate into Neurons and Microglia in both Intact and Status Epilepticus Injured Forebrain. Int J Mol Sci. 2019; 21:181. 10.3390/ijms21010181 31888012 PMC6981466

[43] Uddin MS, Kabir MT, Tewari D, Mamun AA, Mathew B, Aleya L, Barreto GE, Bin-Jumah MN, Abdel-Daim MM, Ashraf GM. Revisiting the role of brain and peripheral Aβ in the pathogenesis of Alzheimer’s disease. J Neurol Sci. 2020; 416:116974. 10.1016/j.jns.2020.116974 32559516

[44] Villalobos Acosta DM, Chimal Vega B, Correa Basurto J, Fragoso Morales LG, Rosales Hernández MC. Recent Advances by In Silico and In Vitro Studies of Amyloid-β 1-42 Fibril Depicted a S-Shape Conformation. Int J Mol Sci. 2018; 19:2415. 10.3390/ijms19082415 30115846 PMC6121414

[45] Yamamoto N, Ishikuro R, Tanida M, Suzuki K, Ikeda-Matsuo Y, Sobue K. Insulin-signaling Pathway Regulates the Degradation of Amyloid β-protein via Astrocytes. Neuroscience. 2018; 385:227–36. 10.1016/j.neuroscience.2018.06.018 29932983

[46] Elia CA, Tamborini M, Rasile M, Desiato G, Marchetti S, Swuec P, Mazzitelli S, Clemente F, Anselmo A, Matteoli M, Malosio ML, Coco S. Intracerebral Injection of Extracellular Vesicles from Mesenchymal Stem Cells Exerts Reduced Aβ Plaque Burden in Early Stages of a Preclinical Model of Alzheimer’s Disease. Cells. 2019; 8:1059. 10.3390/cells8091059 31510042 PMC6770482

[47] Elia CA, Losurdo M, Malosio ML, Coco S. Extracellular Vesicles from Mesenchymal Stem Cells Exert Pleiotropic Effects on Amyloid-β, Inflammation, and Regeneration: A Spark of Hope for Alzheimer’s Disease from Tiny Structures? Bioessays. 2019; 41:e1800199. 10.1002/bies.201800199 30919493

[48] Zhang B, Yeo RW, Tan KH, Lim SK. Focus on Extracellular Vesicles: Therapeutic Potential of Stem Cell-Derived Extracellular Vesicles. Int J Mol Sci. 2016; 17:174. 10.3390/ijms17020174 26861305 PMC4783908

[49] Sørensen SS, Nygaard AB, Christensen T. miRNA expression profiles in cerebrospinal fluid and blood of patients with Alzheimer’s disease and other types of dementia - an exploratory study. Transl Neurodegener. 2016; 5:6. 10.1186/s40035-016-0053-5 26981236 PMC4791887

[50] Kim T, Valera E, Desplats P. Alterations in Striatal microRNA-mRNA Networks Contribute to Neuroinflammation in Multiple System Atrophy. Mol Neurobiol. 2019; 56:7003–21. 10.1007/s12035-019-1577-3 30968343 PMC7216788

[51] Koelsch G. BACE1 Function and Inhibition: Implications of Intervention in the Amyloid Pathway of Alzheimer’s Disease Pathology. Molecules. 2017; 22:1723. 10.3390/molecules22101723 29027981 PMC6151801

[52] Ji Y, Wang D, Zhang B, Lu H. MiR-361-3p inhibits β-amyloid accumulation and attenuates cognitive deficits through targeting BACE1 in Alzheimer’s disease. J Integr Neurosci. 2019; 18:285–91. 10.31083/j.jin.2019.03.1136 31601077

[53] Zhao MY, Wang GQ, Wang NN, Yu QY, Liu RL, Shi WQ. The long-non-coding RNA NEAT1 is a novel target for Alzheimer’s disease progression via miR-124/BACE1 axis. Neurol Res. 2019; 41:489–97. 10.1080/01616412.2018.1548747 31014193

[54] Khalil MN, Choucry MA, El Senousy AS, Hassan A, El-Marasy SA, El Awdan SA, Omar FA. Ambrosin, a potent NF-κβ inhibitor, ameliorates lipopolysaccharide induced memory impairment, comparison to curcumin. PLoS One. 2019; 14:e0219378. 10.1371/journal.pone.0219378 31276550 PMC6611615

[55] De Ferrari GV, Avila ME, Medina MA, Perez-Palma E, Bustos BI, Alarcon MA. Wnt/β-catenin signaling in Alzheimer’s disease. CNS Neurol Disord Drug Targets. 2014; 13:745–54. 10.2174/1871527312666131223113900 24365184

[56] Jia L, Piña-Crespo J, Li Y. Restoring Wnt/β-catenin signaling is a promising therapeutic strategy for Alzheimer’s disease. Mol Brain. 2019; 12:104. 10.1186/s13041-019-0525-5 31801553 PMC6894260

[57] Tapia-Rojas C, Burgos PV, Inestrosa NC. Inhibition of Wnt signaling induces amyloidogenic processing of amyloid precursor protein and the production and aggregation of Amyloid-β (Aβ)_42_ peptides. J Neurochem. 2016; 139:1175–91. 10.1111/jnc.13873 27778356

[58] Vallée A, Vallée JN, Guillevin R, Lecarpentier Y. Riluzole: a therapeutic strategy in Alzheimer’s disease by targeting the WNT/β-catenin pathway. Aging (Albany NY). 2020; 12:3095–113. 10.18632/aging.102830 32035419 PMC7041777

[59] Sun Z, Sun L, Tu L. GABAB Receptor-Mediated PI3K/Akt Signaling Pathway Alleviates Oxidative Stress and Neuronal Cell Injury in a Rat Model of Alzheimer’s Disease. J Alzheimers Dis. 2020; 76:1513–26. 10.3233/JAD-191032 32651311

[60] Kim DY, Choi SH, Lee JS, Kim HJ, Kim HN, Lee JE, Shin JY, Lee PH. Feasibility and Efficacy of Intra-Arterial Administration of Embryonic Stem Cell Derived-Mesenchymal Stem Cells in Animal Model of Alzheimer’s Disease. J Alzheimers Dis. 2020; 76:1281–96. 10.3233/JAD-200026 32597802

[61] Jin Z, Ren J, Qi S. Exosomal miR-9-5p secreted by bone marrow-derived mesenchymal stem cells alleviates osteoarthritis by inhibiting syndecan-1. Cell Tissue Res. 2020; 381:99–114. 10.1007/s00441-020-03193-x 32377874

[62] Si D, Yang P, Jiang R, Zhou H, Wang H, Zhang Y. Improved cognitive outcome after progesterone administration is associated with protecting hippocampal neurons from secondary damage studied *in vitro* and *in vivo*. Behav Brain Res. 2014; 264:135–42. 10.1016/j.bbr.2014.01.049 24518203

[63] Zhang Y, Su P, Liang P, Liu T, Liu X, Liu XY, Zhang B, Han T, Zhu YB, Yin DM, Li J, Zhou Z, Wang KW, Wang Y. The DREAM protein negatively regulates the NMDA receptor through interaction with the NR1 subunit. J Neurosci. 2010; 30:7575–86. 10.1523/JNEUROSCI.1312-10.2010 20519532 PMC6632380

[64] Kim N, Martínez CC, Jang DS, Lee JK, Oh MS. Anti-neuroinflammatory effect of Iresine celosia on lipopolysaccharide-stimulated microglial cells and mouse. Biomed Pharmacother. 2019; 111:1359–66. 10.1016/j.biopha.2019.01.017 30841450

[65] Elmazoglu Z, Rangel-López E, Medina-Campos ON, Pedraza-Chaverri J, Túnez I, Aschner M, Santamaría A, Karasu Ç. Cannabinoid-profiled agents improve cell survival via reduction of oxidative stress and inflammation, and Nrf2 activation in a toxic model combining hyperglycemia+Aβ_1-42_ peptide in rat hippocampal neurons. Neurochem Int. 2020; 140:104817. 10.1016/j.neuint.2020.104817 32781098 PMC7572748

[66] Yang C, Si M, Zhou J. Silencing TRPV4 partially reverses the neurotoxic effects caused by excess Ketamine. J Toxicol Sci. 2021; 46:69–81. 10.2131/jts.46.69 33536391

[67] Fang Y, Shi B, Liu X, Luo J, Rao Z, Liu R, Zeng N. Xiaoyao Pills Attenuate Inflammation and Nerve Injury Induced by Lipopolysaccharide in Hippocampal Neurons In Vitro. Neural Plast. 2020; 2020:8841332. 10.1155/2020/8841332 33014035 PMC7525321

[68] Li JH, Liu S, Zhou H, Qu LH, Yang JH. starBase v2.0: decoding miRNA-ceRNA, miRNA-ncRNA and protein-RNA interaction networks from large-scale CLIP-Seq data. Nucleic Acids Res. 2014; 42:D92–97. 10.1093/nar/gkt1248 24297251 PMC3964941

