# Supplementary material for: Mesenchymal stem cells-derived extracellular vesicles ameliorate Alzheimer’s disease in rat models via the microRNA-29c-3p/BACE1 axis and the Wnt/β-catenin pathway
Source: Aging (Albany NY). 2021 Jun 4;13(11):15285–306. doi: 10.18632/aging.203088 (PMC8221351; doi:10.18632/aging.203088)
Supplement: Supplementary Figures [file aging-13-203088-s001.pdf]

SUPPLEMENTARY FIGURES

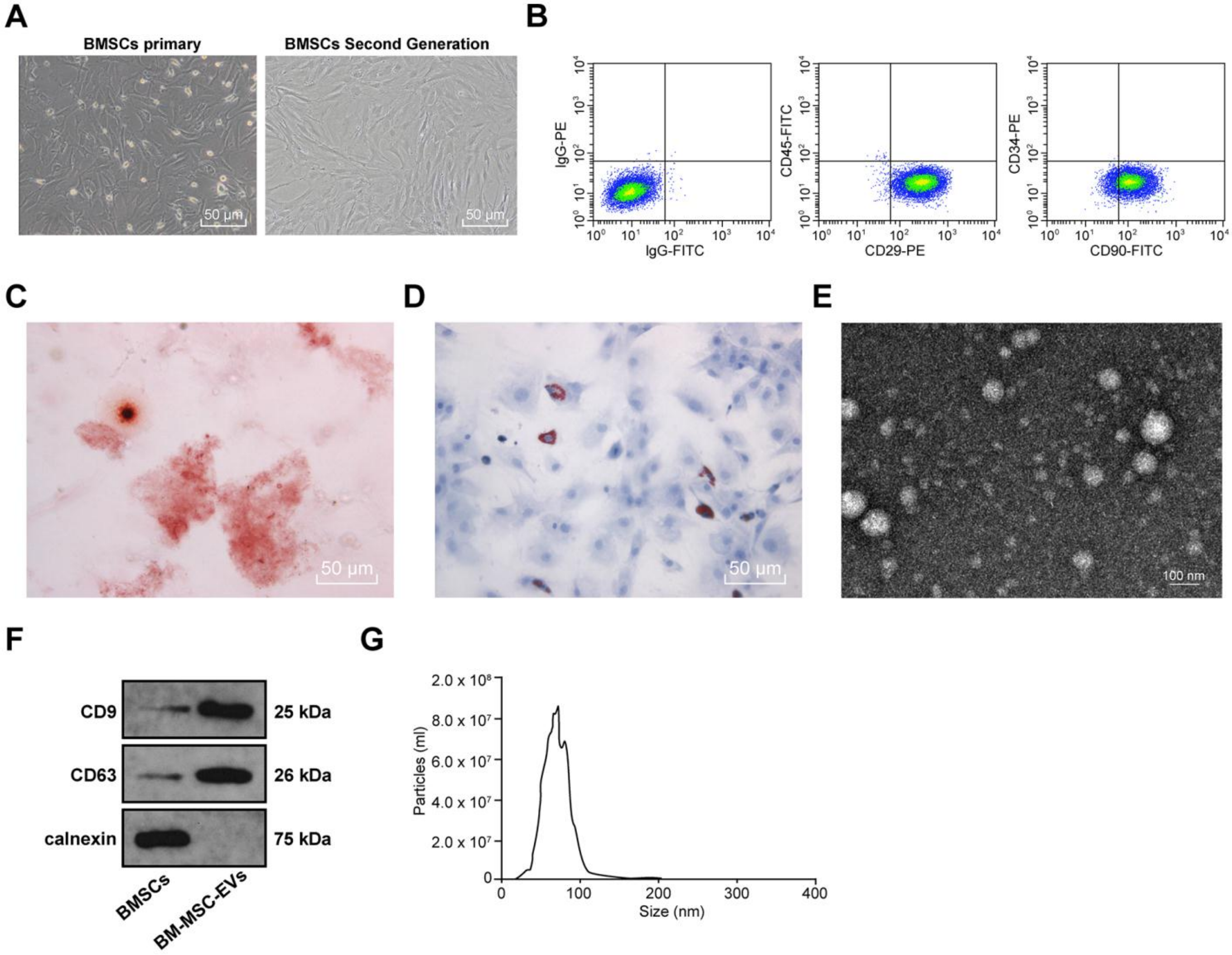

Supplementary Figure 1. Identification of BM-MSCs and EVs. (**A**) The morphology of primary BM-MSCs and BM-MSCs of the 2nd generation was observed under an inverted microscope; (**B**) The expression of CD45, CD29, CD90 and CD34 on the surface of BM-MSCs was detected using flow cytometry; (**C**) Representative images of alizarin red staining of BM-MSCs after 21 days of osteogenic induction; (**D**) Representative images of Oil red O staining of BM-MSCs after 21 days of adipogenic induction; (**E**) The morphology of BM-MSC-EVs was observed under a TEM; (**F**) The expression of CD9, CD63 and calnexin on BM-MSC-EV surface was detected using WB; (**G**) The concentration and particle size of BM-MSC-EVs were measured using NTA. The experiment was repeated three times.

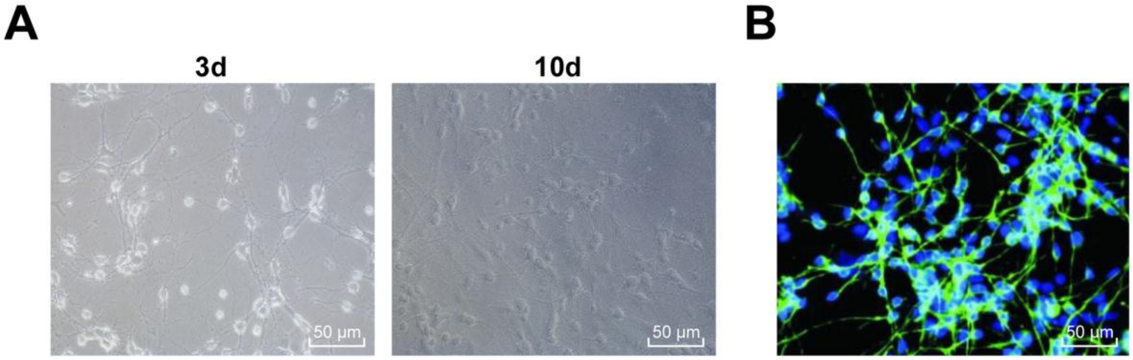

Supplementary Figure 2. Identification of hippocampal neurons. (**A**) The morphology of hippocampal neurons was observed under an inverted microscope; (**B**) Immunofluorescence staining of hippocampal neurons.
